# Characterization of Cleaning and Disinfection Product Use, Glove Use, and Skin Disorders by Healthcare Occupations in a Midwestern Healthcare Facility

**DOI:** 10.3390/buildings12122216

**Published:** 2022-12

**Authors:** Lisa Kobos, Kim Anderson, Laura Kurth, Xiaoming Liang, Caroline P. Groth, Lucy England, A. Scott Laney, M. Abbas Virji

**Affiliations:** 1 Respiratory Health Division, National Institute for Occupational Safety and Health, Morgantown, WV 26505, USA; 2 Department of Epidemiology and Biostatistics, School of Public Health, West Virginia University, Morgantown, WV 26505, USA

**Keywords:** cleaning product exposure, healthcare, personal protective equipment, glove use, skin disorders

## Abstract

Healthcare facility staff use a wide variety of cleaning and disinfecting products during their daily operations, many of which are associated with respiratory or skin irritation or sensitization with repeated exposure. The objective of this study was to characterize the prevalence of cleaning and disinfection product use, glove use during cleaning and disinfection, and skin/allergy symptoms by occupation and identify the factors influencing glove use among the healthcare facility staff. A questionnaire was administered to the current employees at a midwestern Veterans Affairs healthcare facility that elicited information on cleaning and disinfection product use, glove use during cleaning and disinfection, skin/allergy symptoms, and other demographic characteristics, which were summarized by occupation. The central supply/environmental service workers (2% of the total survey population), nurses (26%,), nurse assistants (3%), and laboratory technicians (5%) had the highest prevalence of using cleaning or disinfecting products, specifically quaternary ammonium compounds, bleach, and alcohol. Glove use while using products was common in both patient care and non-patient care occupations. The factors associated with glove use included using bleach or quaternary ammonium compounds and using cleaning products 2–3 or 4–5 days per week. A high frequency of glove use (≥75%) was reported by workers in most occupations when using quaternary ammonium compounds or bleach. The use of alcohol, bleach, and quaternary ammonium compounds was associated with skin disorders (*p* < 0.05). These research findings indicate that although the workers from most occupations report a high frequency of glove use when using cleaning and disinfection products, there is room for improvement, especially among administrative, maintenance, and nursing workers. These groups may represent populations which could benefit from the implementation of workplace interventions and further training regarding the use of personal protective equipment and the potential health hazards of exposure to cleaning and disinfecting chemicals.

## 1. Introduction

The healthcare industry is one of the largest domestic employment sectors in the United States, employing slightly over 20 million individuals in 2020, or approximately 14% of all working adults [1]. The industry includes occupations that directly provide healthcare services as well as those that provide supporting services. Healthcare workers are reported to have an elevated prevalence of asthma and respiratory symptoms. Data from the 2013 Behavioral Risk Factor Surveillance System collected from 21 states show that healthcare and healthcare support workers had among the highest prevalence of asthma of the occupations surveyed at 10.7% and 12.4%, respectively (compared to 7.7% among the general population) [2]. Healthcare workers who perform cleaning and disinfection tasks have a higher prevalence of respiratory symptoms and asthma [3,4]. In addition, healthcare workers have an increased risk of developing eczema or dermatitis [5] when they are performing “wet work”, defined as activities that involve immersing one’s hands in liquids for >2 h per shift, wearing waterproof (occlusive) gloves for a corresponding amount of time, or washing one’s hands > 20 times per shift [6–9]. Wearing occlusive gloves can also cause irritation due to friction or an accumulation of sweat and oils on the workers’ hands [8]. The human and economic costs of these conditions are substantial, costing the healthcare system billions of dollars annually, including the cost of lost work time from absenteeism due to asthma [10,11]. For the workers in the healthcare industry, a decrease in productivity has a magnified impact: not only does their illness affect them directly, but it could also lead to a decline in facility operations and patient care.

Healthcare facilities use a wide range of cleaning and disinfection products to meet infection control guidelines and keep their staff and patients safe from healthcare-associated infections; however, the use of cleaning and disinfection products such as bleach, glutaraldehyde, and chlorhexidine in healthcare settings has been associated with respiratory symptoms, including asthma-like symptoms, and skin conditions [12–14]. Respiratory symptoms have also been associated with the use of most major classes of cleaning and disinfecting products including chlorine-based compounds [15,16], ammonia [16], quaternary ammonium compounds [15,17], peroxygen compounds [18–20], enzymatic cleaners [15,21], detergents [16,22], high-level disinfectants and sterilants [15,16], and their functional or aesthetic additives such as ethanolamines and terpenes [23–26].

Previous observational studies have shown that nurses spend substantial amounts of time performing wet work, which is associated with skin disorders. Nurses can spend on average a quarter of their shift with their hands being exposed to water, chemicals, foodstuffs, or gloves [6,27,28]. However, specific wet work activities associated with the skin disorders have not been well described. Moreover, glove use in general among healthcare workers performing patient care or non-patient care tasks is often suboptimal, with glove use compliance at or below 75%, and with some facilities as low as 44% [29–32]. These studies and others report on the adherence of glove use for infection control, though the data are lacking on glove use to protect against skin exposure to cleaning chemicals. Two studies were identified in the literature that investigated glove use for skin protection from the use of cleaning products. Henn et al. reported that 9% of participants did not always wear gloves when handling high-level disinfectants, while Humann et al. reported that the proportion of time of glove use increased when a sensitizer was used, although this change varied based on occupation [33,34]. However, little is known regarding the specific conditions under which protective gloves are used.

The objective of the current study was to characterize the prevalence of cleaning and disinfection product use, glove use during cleaning and disinfection product use, hand hygiene, and specified skin conditions in both patient care and non-patient care occupations at a Veteran Affairs (VA) medical center in the midwestern United States.

## 2. Materials and Methods

### 2.1. Survey Methodology

A questionnaire was administered to collect information on respiratory and skin health, demographic and occupational characteristics, and workplace exposures. The questionnaire was adapted from survey instruments previously used at this Veterans Affairs (VA) medical center and other healthcare facilities [4,35] and is described in detail elsewhere [36,37]. The survey questions require mostly binary or multiple-choice responses, and the questionnaire (with identifying information removed) is available to view as a supplementary file to this publication (File S1., Healthcare Facility Questionnaire). Briefly, work exposure questions included a module on cleaning and disinfecting fixed surfaces, equipment, or medical instruments. The participants were asked about hand hygiene, the duration and frequency of product use on surfaces or on instruments, and glove use during cleaning and disinfection product use. The health questions asked about the participant’s history of rubber allergies and skin disorders (e.g., hives in the past 12 months, rash at work related to glove use in the past 12 months) and if they had ever experienced atopic dermatitis. The participants were also asked questions to determine their allergic status, with “positive” being defined as them ever having atopic dermatitis, allergic eye symptoms, or seasonal allergic rhinitis. Current asthma was defined as a positive response to each of the following three questions: “Have you ever had asthma?”, “Was your asthma confirmed/diagnosed by a doctor or a healthcare professional?”, and “Do you still have asthma?”.

The survey was open to all current facility employees during July 2012–August 2014. Advertisements, employee email, and in-person outreach were used to encourage employee participation. The survey was open to all current employees of the facility with no undue barriers to participation. The internet-based Research Electronic Data Capture (REDCap) system was used to collect, enter, store, and manage the survey data. The information was entered directly into REDCap by the participants when they were completing the computer-based questionnaire. If the participant completed the survey using a paper questionnaire, the data were subsequently entered by two study coordinators at the VA medical center, who then sent the anonymized data to the National Institute of Occupational Safety and Health (NIOSH).

### 2.2. Data Analysis

Frequencies were calculated to determine the prevalence of cleaning and disinfection product use, skin disorders/allergy symptoms, and glove use by occupation and occupational category. The data on race were collapsed into two levels representing White race and non-White race, which included African-American or Black, Asian, American Indian or Alaska Native, Native Hawaiian or Pacific Islander, and Other due to there being a small sample size. Additionally, age, number of hours worked per week, and total years worked in healthcare were also grouped due to there being a small sample size. The occupations were grouped into three categories (administration, patient care, and non-patient care) to evaluate higher level patterns in product use, glove use, or skin disorders. The patient care occupations included: respiratory therapists, licensed practical nurses, nurses, nursing assistants, other nurses, and other technicians (this included dental assistants and surgical technicians). The non-patient care occupations included: central supply workers, environmental service workers, laboratory technicians (including biomedical engineers or technicians), maintenance, and pharmacists or pharmacy technicians. Univariate and multiple logistic regression models were utilized to examine the associations between glove use, cleaning and disinfecting product use, hand hygiene, and worker characteristics. Univariate and multiple regression models were also used to determine the association of product use with skin, allergy, and any skin or and allergy disorders, and allergic reactions in isolation and in combination. The “skin disorders” category was classified as those having ever experienced atopic dermatitis or a rash in the past 12 months. The “allergic reactions” category was classified as having hives in the past 12 months, allergies to rubber, having ever had an allergic reaction during a dental or OBGYN exam, or ever having experienced a life-threatening allergic reaction. The data were analyzed using JMP 15 (version 15.1.0, SAS Institute Inc., Cary, NC, USA), SAS (version 9.4, SAS Institute Inc.), RStudio version 1.4.1717, and R version 4.1.1 (RStudio, Boston, MA, USA). A *p*-value < 0.05 was used to determine statistical significance.

### 2.3. Human Subjects Review Board

The survey protocol for this study was approved by the VA’s human subjects committee and NIOSH’s human subjects review board. By completing the questionnaire, the participants indicated consent to take part in the survey.

## 3. Results

At the conclusion of the survey period, a total of 559 questionnaires were retained after excluding 47 of them due to them having incomplete data or participant’s refusal to consent, and excluding 81 duplicate records. During the survey period, the average annual total number of employees at the facility was 3591, yielding an effective participation rate of 15.7%; demographic information on the source population was not available. A previous study on this worker population that reported on the association of cleaning product use and tasks with respiratory symptoms and asthma focused only on participants in the main hospital building, which is a subset of those included in the current study [36].

### 3.1. Occupation and Product Use by Race and Sex

Most of the study participants were female (77%), White (88%), and worked 40–49 h per week (85%), with a median age of 49 years (range: 20–73 years) and median of 19 years of healthcare experience (range: <1–50 years) (Table 1).

The most common occupations were nurses (*n* = 149), administrators (*n* = 123), and technicians (other) (*n* = 93). Some differences by race and sex existed in certain occupations, though it should be noted that due to the small sample size of some occupations and the low survey response rate, these patterns should be interpreted with caution (Table 2).

The non-patient care workers had a slightly higher proportion of non-White individuals compared with the total number of survey respondents (20% for non-patient care, 12% total). Central supply/environmental service workers, maintenance workers, and nursing assistants were 40%, 35%, and 37.5% non-White. Additionally, 70% of the central supply/environmental services workers and 65% of the maintenance workers were male, compared to 23% of the total pool of survey respondents. Patient care occupations, including nurses and technicians (other), were predominantly White (94% and 95%, respectively) compared to the total pool of survey respondents (88%). Additionally, 38% of the respiratory therapists were male compared to the total pool of survey respondents which were 23% male. The frequencies of product use by race and sex were similar to the general survey population, except for the use of alcohol and bleach for instrument disinfection (30% and 33%, respectively), which was more common among males relative to their prevalence within the total pool of survey respondents (23%).

### 3.2. Product Use

Overall, the cleaning and disinfection products with the highest frequency of reported use for the total worker population were quaternary ammonium compounds (on surfaces) (28%), alcohol (on surfaces) (26%), and bleach (on surfaces) (18%) (Table 3).

All of the occupations primarily used cleaning products to clean surfaces. The non-patient care workers with the highest reported frequency of alcohol and bleach use on surfaces were central supply/environmental service workers (20% and 80%, respectively) and laboratory technicians (60% and 60%, respectively).

### 3.3. Glove Use during Cleaning and Disinfection Product Use

Overall, the reported frequencies of using gloves while using a chemical product ranged from 87% for quaternary ammonium compounds to 47% for glass cleaner (Table 4).

The non-patient care occupations generally had the highest frequencies of glove use while using cleaning chemicals, ranging from 71% while alcohol on medical instruments to 97% while using bleach on surfaces. The central supply/environmental service workers and laboratory technicians exemplified this, with most of them (75% or more) reporting glove use while using any cleaning products. Glove use was less common for almost all cleaning products among the administrative workers. Among the patient care occupations the highest frequencies of glove use were reported for using quaternary ammonium compounds on surfaces (90%) and bleach on medical instruments (88%), and the lowest frequencies were reported for using glass cleaners (45%). Specifically, nurses, nursing assistants and other nurses all reported glove use at or above 75% when cleaning medical instruments with bleach or alcohol. All of the patient care occupations reported high frequencies of glove use with quaternary ammonium compounds, ranging from 75% for licensed practical nurses to 100% for nursing assistants. Both the patient care and non-patient care categories reported high frequencies of using gloves while using quaternary ammonium compounds (90% and 93%), though glove use was lower for the administrative workers at 56%.

### 3.4. Factors Associated with Glove Use

Univariate models were fit to explore associations between glove use, cleaning and disinfecting product use, hand hygiene, and participant characteristics (Table 5).

Elevated odds ratios (OR) were observed for wearing gloves during the use of bleach (OR: 9.0, 95% confidence interval (CI): 2.6, 31.9) and quaternary ammonium compounds (OR: 13.4, CI: 3.9, 46.4) compared to the reference group of alcohol use. Those who used cleaning products 2–3 days per week (OR: 2.5, CI: 1.1, 5.7) or 4–5 days per week (OR: 3.9, CI: 1.9, 8.2) had higher odds of wearing gloves than the reference group, who only used products 1 day or less per week. Both the patient care (OR: 8.2, CI: 1.8, 36.8) and non-patient care (OR: 21.3, CI: 3.6, 125.9) occupations had significantly higher odds of glove use than those in administration. Elevated odds ratios for glove use were also obtained for those who used hand sanitizer 4–10 or more than 10 times per day compared to those who used hand sanitizer less than once daily, although this was non-significant (Table 5). In the multiple logistic regression model, product use and occupation were significant predictors of glove use. Specifically, the patient care and non-patient care categories had odds ratios of 9.1 (CI: 1.3, 63.0) and 43.7 (CI: 3.6, 523.5), respectively, when they were compared to the reference group of administrative workers. The use of bleach (OR: 9.2, CI: 2.8, 30.1) and quaternary ammonium compounds (OR: 11.5, CI: 3.6, 36.3) was significantly associated with glove use when compared to alcohol use.

### 3.5. Skin Disorders and Rubber Allergies

The frequencies of skin disorders and rubber allergies are summarized by sex, race, age, occupation, the total years worked in healthcare, the number of hours worked per week, hand sanitizer use, handwashing frequency, allergic status, and whether the participants reported having current asthma (Table 6).

Nurses made up 26% of the total study population, but they comprised 44% of workers who reported work rashes. The respondents in the “Other Technician” category made up only 11% of all of the cases of atopic dermatitis despite representing 17% of the total survey population. The female workers represented 77% of the survey population, but 86% of participants who reported hives in the last 12 months were female, and 100% of participants who reported having a rubber allergy were female. Those between 20–39 years of age were more likely to report experiencing hives or a rash at work as a result of glove use. The workers with a longer tenure (10–29 years) were more likely to have experienced a rash at work as a result of glove use or a rubber allergy. Among those who experienced rashes at work due to gloves, 67% and 50% of them reported washing or sanitizing more than ten times per day, respectively, despite only 37% and 30% of the total study population reporting those frequencies of hand hygiene. Additionally, a higher frequency of hives in the last 12 months was reported among those who washed (but not those who sanitized) their hands this frequently. The participants with asthma or who had a positive allergic status were disproportionately likely to suffer from all the skin disorders listed, relative to their prevalence among the overall study population. Those with a positive allergic status were more likely to report the presence of both asthma and a skin disorder; with combined atopic dermatitis and asthma at 9% for those with positive status vs. 0% for those with negative status, and hives at 7% and 1%.

**Figure 1.**
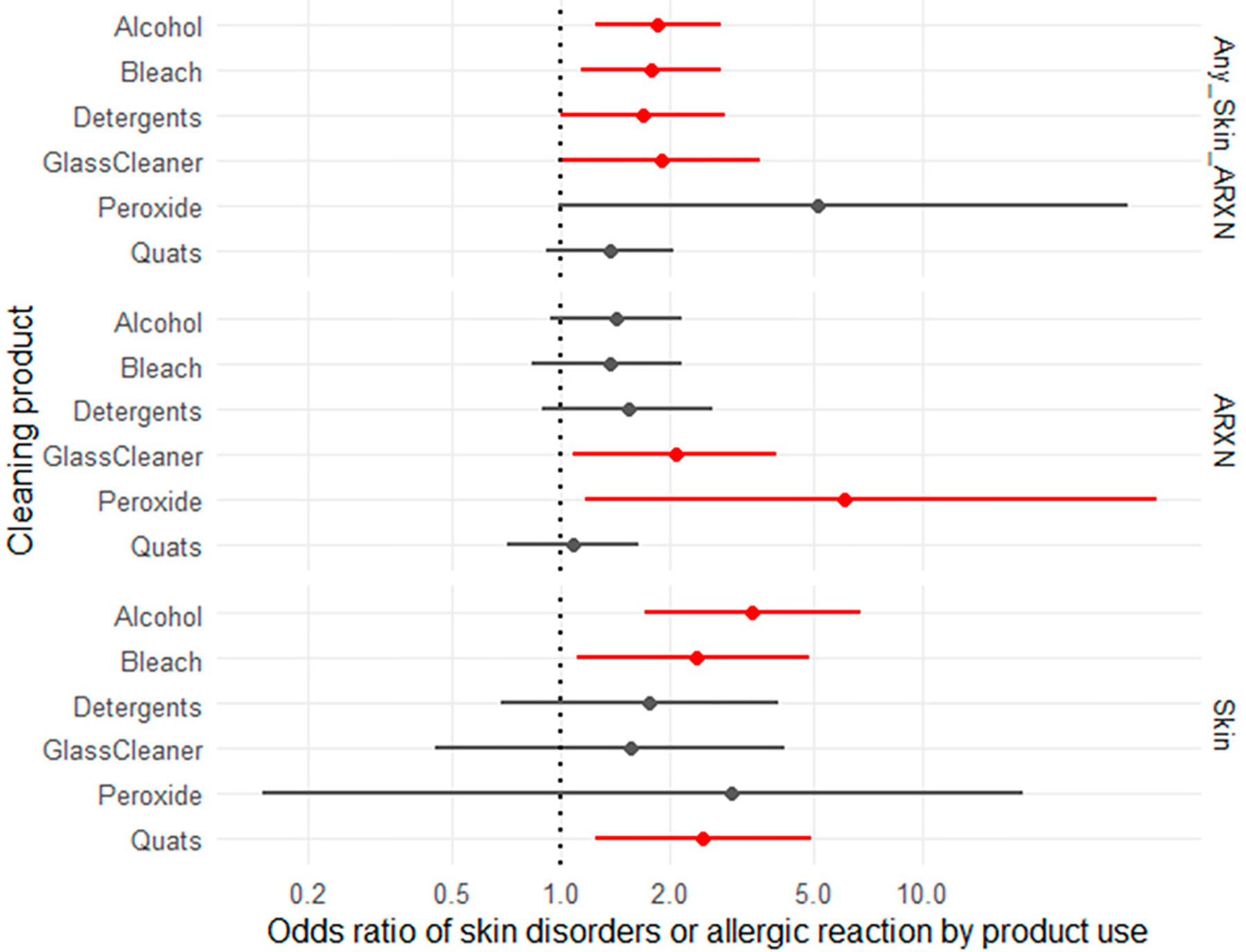
Forest plot showing the odds ratios and confidence intervals for skin disorders and allergy symptoms by the use of specific products. Red indicates an odds ratio that was deemed to be significant, while black indicates an odds ratio deemed non‐significant.

Univariate models were fit to evaluate the associations between cleaning product use and (1) skin disorders, (2) allergic reactions, or (3) any skin disorder or allergic reaction (Figure 1). The odds of developing any skin disorder or allergic reaction were significantly elevated with alcohol, bleach, detergent, or glass cleaning product use, with use of glass cleaning products having the strongest association (OR: 1.86, 95% CI: 1.25–2.77; OR: 1.79, 95% CI: 1.14–2.8; OR: 1.69, 95% CI: 1–2.84; OR:1.91, 95% CI: 1–3.59, respectively). Skin disorders were strongly associated with using cleaning products with alcohol, bleach, or quaternary compounds (OR: 3.4, 95% CI: 1.72–6.81; OR: 2.39, 95% CI: 1.12–4.87; OR:2.49, 95% CI: 1.25–4.94, respectively) compared to those who did not use cleaning products containing those compounds. The respondents who used cleaning products containing glass cleaners or peroxide were from 2-fold to 6-fold more likely to report allergic reactions compared to the respondents who did not use those products (OR: 2.08, 95% CI: 1.08–3.94; OR: 6.12, 95% CI: 1.18–44.47). Only the univariate models were significant. When product use was added to the full model containing occupation, gender, and race, product use was no longer significantly associated with any of the health outcomes that were evaluated.

## 4. Discussion

Healthcare workers have a higher prevalence of asthma and skin disorders than the general population does due to a number of factors, including using cleaning and disinfection products, the performance of wet work, and the frequency and duration of high-exposure cleaning tasks [6–9,13,15,16,37]. In this study, we evaluated the prevalence of cleaning and disinfection product use, glove use, and skin disorders by occupation and other worker characteristics and hand hygiene practices. The central supply/environmental service workers, nurses, nurse assistants, and laboratory technicians had the highest prevalence of cleaning product use, specifically quaternary ammonium compounds, bleach, and alcohol. Glove use while using cleaning products was prevalent in both patient care and non-patient care occupations, albeit less frequently than desirable. Other studies have also reported suboptimal frequencies of proper glove use in general among healthcare workers [29–32,38]. While the issues of skin/respiratory conditions and a low level of personal protective equipment (PPE) compliance among healthcare workers are well-established, limited research has been performed regarding specific factors which may be associated with glove use or occupational illness. In this study, the workers who used bleach or quaternary ammonium compounds or used cleaning products 2–3 or 4–5 days per week were more likely to use gloves than those who used alcohol to clean or worked 1 or fewer days per week. Additionally, participants who were in the nursing occupation, female, or washed or sanitized their hands more than 10 times per day reported a higher prevalence of skin disorders and rubber allergies. In comparison to previous studies which only evaluated the frequency of glove use, the targeted approach of this study provides more in-depth insights regarding glove use, hand hygiene, worker characteristics, and work-related skin conditions. Understanding the factors associated with glove use and skin disorders may provide opportunities to target intervention measures.

### 4.1. Product Use

In this study, we found that the most common cleaning products used on surfaces were quaternary ammonium compounds, alcohol, and bleach. Other studies on the use of cleaning products in U.S. healthcare settings have yielded similar results. One study of approximately 300 healthcare workers demonstrated that isopropyl alcohol was the most frequently used chemical among those who were regularly exposed to cleaning products, with over 70% of the respondents stating that they used the chemical at least once per week. Quaternary ammonium compounds were the second most frequently used product (at approximately 46%), followed closely by bleach (at approximately 45%) [13]. In a separate survey of 3650 healthcare professionals (physicians, nurses, respiratory therapists, and occupational therapists), 55.5% of them reported regular exposure to bleach [16]. Another study examining cleaning and disinfection product use by healthcare occupation determined that alcohol was the most widely used product ingredient, being used for at least five minutes per shift by 10 of the 14 occupations that were surveyed [39].

### 4.2. Glove Use

The surveyed workers were more likely (relative to their respective reference groups) to report using gloves if they used bleach or quaternary ammonium compounds, used cleaning products 2–3 or 4–5 days weekly, or were in patient care or non-patient care occupations. The research on the predictors of glove use associated with using cleaning products is limited, and it is largely unknown whether these findings represent an average healthcare facility. Previous investigations have found that glove use during product use does vary among occupations in healthcare settings, with one study finding that endoscopy technicians had the highest proportion of glove use during contact with and/or use of liquids [34]. Respiratory therapists and laboratory technicians had among the lowest proportion of glove use during contact with and/or use of liquids. A survey of approximately 5000 healthcare professionals in the United States found that 9% of them did not always wear PPE while handling high-level disinfectants [33]. Among those who reported direct skin exposure to high-level disinfectants, 33% of them did not always wear gloves. Glove use to comply with infection control requirements in healthcare settings was reported to vary by occupation [40] and work location e.g., surgical sites and operating rooms showed the highest compliance with glove use [41,42].

### 4.3. Differences in Product Use, Skin Disorders, and Rubber Allergy by Sex

The results of the survey demonstrated that a larger proportion of females reported using quaternary ammonium compounds on surfaces, while a larger proportion of males reported the use of alcohol and bleach on medical instruments. This is likely due to the unequal representation in various professions, with females being overrepresented in the nurse occupation, and nurses having among the highest reported frequency of quaternary ammonium compound use. Similarly, males comprise a larger proportion of central supply or environmental service workers, and this occupation had the highest reported frequency of bleach and alcohol use while cleaning medical instruments. Females reported a higher prevalence of hives and rubber allergy than males. This is unsurprising, as after puberty, females are generally more susceptible to allergies than males, due at least in part to the effects of hormones on the immune system [43–46]. The disproportionate representation of females in nursing, including nursing assistants, might also contribute to this disparity; those in nursing are more likely to experience skin conditions and allergies related to work [47–49]. The significant amount of time nurses spend performing wet work likely contributes to their susceptibility, as an established link exists between the performance of wet work and eczema or dermatitis [6–8].

### 4.4. Skin Disorders and Allergy Symptoms

The survey results found that the workers with a positive allergic status (i.e., having experienced atopic dermatitis, allergic eye symptoms, and/or seasonal allergic rhinitis) were likely to report hives, rash, and a rubber allergy. The ailment with the lowest frequency among those with a positive allergic status was hives, at 78%. Previous research on the connection between skin, allergy, and respiratory symptoms has shown that they are often associated with one another, as all commonly have a component of immunoglobulin E (IgE)-mediated reactions, also known as type-1 hypersensitivity reactions. Past investigations have found that higher levels of serum IgE are associated with respiratory and skin symptoms, such as airway hyperresponsiveness, allergic sensitization, asthma, rhinoconjunctivitis, and contact urticaria [50,51]. A survey of 453 individuals determined that among those occupationally sensitized to a specific allergen (laboratory animals), 16% of them reported having skin disorders, rhinitis, and asthma [52]. This overlap was less than 2% for the non-sensitized respondents, and it was 5% among those who were sensitized by non-workplace exposures. Consistent with the findings reported in the literature, in the present study, a larger percentage of participants with allergies reported having both asthma and a skin disorder (atopic dermatitis or hives) than those who did not have allergies. Other technicians had a lower prevalence of atopic dermatitis, possibly due to their low frequency of chemical exposure. Only 26% of the other technicians reported using alcohol on surfaces, and only 21% reported using quaternary ammonium compounds. The reported exposures to other chemicals were even lower, ranging from 7% for bleach on surfaces to 1% for alcohol on medical instruments.

Chemical exposure itself was determined to be significantly associated with skin and allergy symptoms. Specifically, alcohol, bleach, and quaternary compound use was associated with skin disorders. These findings are largely in concordance with those of other studies, demonstrating a connection between skin problems and the use of alcohol, bleach, and quaternary compounds. Bleach has been previously associated with hand dermatitis in healthcare workers [13]. Topical application of alcohol is known to significantly reduce the skin’s hydration, a key factor in protecting the skin barrier and preventing conditions such as dermatitis and eczema [53–56]. Multiple cases of contact dermatitis as a result of occupational exposure to quaternary ammonium compounds have been documented, both within and outside of a healthcare setting [57–59]. Additionally, both peroxide and glass cleaner were significantly associated with allergy symptoms, a finding that is not supported by any previous research that the authors are aware of. When skin disorders and allergy symptoms were combined into a single illness category, significant associations were observed for alcohol, bleach, glass cleaner, and detergent use, but not for peroxide and quaternary ammonium compounds. Detergent use is well documented to be a risk factor for allergic dermatitis in healthcare workers [60–62]. Overall, this investigation, in combination with the others cited previously, demonstrates a strong connection between skin, allergy, and respiratory illness, particularly among those exposed in an occupational setting. However, the relationship with cleaning product use is confounded by other factors.

### 4.5. Limitations

The survey was limited in scope to a single healthcare facility, limiting its generalizability to the overall population of healthcare workers, though this population and study at large may serve as a representative example for future investigations. The findings presented here may inform the content and structure of future questionnaires, the execution of survey collection in the workplace, and ultimately, the development and implementation of interventional strategies. This survey was performed prior to the COVID-19 pandemic, meaning that the work practices in healthcare settings have likely changed substantially since these data were collected. The rate of survey participation was relatively low at 15%, but this is similar to other questionnaire surveys administered in other healthcare settings [4]. Nevertheless, the survey results may not be reflective of the workforce as a whole. The number of non-White participants was low enough that the data were necessarily combined for analyses, which may have resulted in the loss of nuance in the study findings. Regarding rashes at work, the question only accounted for rashes that the workers attributed to gloves without taking into account other possible causes; confirming gloves as the cause was beyond the scope of this paper. For the same reason, hives were not confirmed as being related to dermal exposures or exposures that occurred at work. The survey was self-reported, and it was thus subject to social-desirability bias (i.e., underreporting of “bad” or undesirable behavior). Due to the cross-sectional design of the study, causal inferences could not be made. For the same reason, we were unable to determine the effects of exposure over time and likely did not capture conditions that occurred for only a short time.

### 4.6. Potential Solutions

While encouraging glove use among workers does represent a potential avenue for decreasing the incidence of occupational illness, other options should be considered. In fact, excessive glove use may be an obstacle in terms of implementing appropriate hand hygiene practices, as healthcare workers may wear gloves in lieu of washing or disinfecting their hands [63]. Glove use should be encouraged, but care should be taken that it is not emphasized to the exclusion of other worker safety measures. While the elimination of cleaning chemicals in a healthcare setting is clearly unfeasible, substituting gentler cleaning chemicals could reduce worker’ exposure. Workers should also be encouraged to change their gloves frequently to avoid occlusive glove exposure for extended durations. Supervisory and managerial staff may arrange or conduct periodic training to ensure that proper glove use practices remain familiar. Other interventions that may help to reduce the incidence of skin disorders include the use of gentler alcohol-based gels rather than harsher soaps and the use of conditioning creams, though the evidence of the efficacy of these methods is relatively limited [1,64]. Ideally, this should be coupled with education programs and information campaigns, as these have previously resulted in improved hand hygiene more so than just increasing the availability of interventions [65]. Further, given that regularly wearing gloves for extended periods of time has been linked with skin conditions such as eczema [7], glove use should be limited to situations where it is appropriate and necessary. The workers should be provided with the tools and education they need to understand when occlusive gloves are and are not needed.

## 5. Conclusions

In this study, we surveyed the workers at a healthcare facility to assess the prevalence of cleaning and disinfection product use, glove use, skin disorders and rubber allergy, and their association with occupation, demographics, and hand hygiene. Central supply or environmental service workers, nurses, nurse assistants, and laboratory technicians had the highest prevalence of cleaning and disinfection product use, specifically quaternary ammonium compounds, bleach, and alcohol. Certain workers, specifically those who reported using bleach or quaternary ammonium compounds, using cleaning and disinfection products 2–3 or 4–5 days per week, and those in patient care or non-patient care occupations were more likely to use gloves relative to the reference groups. Further, an assessment of the self-reported skin conditions demonstrated a higher prevalence for nurses, females, and those who washed or sanitized their hands more than 10 times per day compared to other occupations, males, and those who washed or sanitized their hands less frequently, respectively. These findings indicate that, due to a combination of product use, glove use, and work tasks, certain groups are potentially more susceptible to the development of an occupational skin illness. The results of this study may be utilized in the development and implementation of targeted intervention strategies to minimize exposure and maintain skin health.

## Supplementary Material

Supplementary Materials

## Figures and Tables

**Table 1.** Participant characteristics, N (%).

Demographics	N (%)
**Sex**	
Male	128 (23%)
Female	431 (77%)
**Age**	
20–39	158 (28%)
40–49	123 (22%)
50–59	206 (37%)
60+	72 (13%)
**Race**	
White	493 (88%)
African American	44 (8%)
Other	12 (2%)
Unspecified	10 (2%)
**Total Years Worked**	
0–9	167 (30%)
10–19	118 (21%)
20–29	136 (24.4%)
30–39	107 (19.2%)
40+	30 (5.4%)
**Hours/Week**	
<20	12 (2%)
20–29	27 (5%)
30–39	22 (4%)
40–49	473 (85%)
>49	23 (4%)

Characteristics of survey participants, specifically sex, age, race, total years worked in healthcare, and number of hours worked per week. Category frequencies are expressed using numbers (*n*) and percentages.

**Table 2.** Distribution of occupation and cleaning products used by race and sex, N (%).

	Race		Sex	
Occupation (N, %)	White	Non-White	Male	Female
Total (559)	493 (88%)	66 (12%)	128 (23%)	431 (77%)
Administration (123, 22%)	106 (86%)	17 (14%)	31 (25%)	92 (75%)
Non-Patient care * (84, 15%)	67 (80%)	17 (20%)	35 (42%)	49 (58%)
Central Supply or Environmental Service Worker (10, 2%)	6 (60%)	4 (40%)	7 (70%)	3 (30%)
Lab Technician (30, 5%)	26 (87%)	4 (13%)	7 (23%)	23 (77%)
Maintenance (26, 5%)	17 (65%)	9 (35%)	17 (65%)	9 (35%)
Pharmacist or Pharmacy Technician (14, 3%)	14 (100%)	-	2 (14%)	12 (86%)
Patient care (341, 61%)	312 (91%)	29 (9%)	57 (17%)	284 (83%)
Licensed Practical Nurse (18, 3%)	16 (89%)	2 (11%)	3 (17%)	15 (83%)
Nurse (149, 26%)	140 (94%)	9 (6%)	19 (13%)	130 (87%)
Nursing Assistant (16, 3%)	10 (62.5%)	6 (37.5%)	1 (6%)	15 (94%)
Other Nurse (57, 10%)	51 (89.5%)	6 (10.5%)	12 (21%)	45 (79%)
Other Technician (93, 17%)	88 (95%)	5 (5%)	19 (20%)	74 (80%)
Respiratory Therapist or Technician (8, 2%)	7 (87.5%)	1 (12.5%)	3 (38%)	5 (62%)
Other (11, 2%)	8 (73%)	3 (27%)	5 (45%)	6 (55%)
**Product Use**				
Detergent	62 (89%)	8 (11%)	18 (26%)	52 (74%)
Glass Cleaner	38 (88%)	5 (12%)	10 (23%)	33 (77%)
Quaternary Ammonium Compounds	142 (91%)	14 (9%)	27 (17%)	129 (83%)
Alcohol (Surface)	129 (90%)	14 (10%)	27 (19%)	116 (81%)
Alcohol (MI)	18 (90%)	2 (10%)	6 (30%)	14 (70%)
Bleach (Surface)	90 (90%)	10 (10%)	19 (19%)	81 (81%)
Bleach (MI)	13 (87%)	2 (13%)	5 (33%)	10 (67%)

Occupations of the survey participants and cleaning product use, distributed by race and sex. Category frequencies are expressed using numbers (*n*) and percentages. A value of—indicates a reported result of 0 (0). MI indicates medical instruments.

* For the purposes of this analysis, biomedical engineers/technicians (*n* = 4) were classified as non-patient care workers.

**Table 3.** Distribution of occupation by cleaning products used, N (%).

	Products, N (%)						
Occupation (N)	Detergent *n* = 70	Glass Cleaner *n* = 43	Quaternary Ammonium Compounds (Surface) *n* = 156	Alcohol (Surface) *n* = 143	Alcohol (MI) *n* = 20	Bleach (Surface) *n* = 100	Bleach (MI) *n* = 15
Total (559)	70 (13%)	43 (8%)	156 (28%)	143 (26%)	20 (4%)	100 (18%)	15 (3%)
Administration (123)	6 (5%)	9 (7%)	16 (13%)	12 (10%)	1 (0.8%)	7 (6%)	-
Non-Patient care * (84)	24 (29%)	12 (14%)	28 (33%)	28 (33%)	7 (8%)	32 (38%)	7 (8%)
Central Supply or Environmental Service Worker (10)	7 (70%)	6 (60%)	8 (80%)	2 (20%)	2 (20%)	8 (80%)	2 (20%)
Lab Technician (30)	8 (27%)	1 (3%)	15 (50%)	18 (60%)	2 (7%)	18 (60%)	2 (7%)
Maintenance (26)	8 (31%)	4 (15%)	4 (15%)	5 (19%)	1 (4%)	4 (15%)	1 (4%)
Pharmacist or Pharmacy Technician (14)	1 (7%)	-	1 (7%)	2 (14%)	1 (7%)	1 (7%)	1 (7%)
Patient care (341)	40 (12%)	22 (6%)	112 (33%)	103 (30%)	12 (4%)	61 (18%)	8 (2%)
Licensed Practical Nurse (18)	-	1 (6%)	4 (22%)	4 (22%)	-	2 (11%)	-
Nurse (149)	24 (16%)	12 (8%)	70 (47%)	57 (38%)	7 (5%)	38 (26%)	4 (3%)
Nursing Assistant (16)	3 (19%)	2 (13%)	2 (13%)	6 (38%)	3 (19%)	3 (19%)	2 (13%)
Other Nurse (57)	8 (14%)	3 (5%)	13 (23%)	9 (16%)	1 (2%)	11 (19%)	1 (2%)
Other Technician (93)	5 (5%)	4 (4%)	20 (21%)	24 (26%)	-	6 (6%)	-
Respiratory Therapist or Technician (8)	-	-	3 (38%)	3 (38%)	1 (13%)	1 (13%)	1 (13%)
Other (11)		-		-		-	

Percentage of workers in each occupation or category who use a specific chemical, expressed as a percentage of total survey participants in that occupation. Frequency of affirmative responses expressed using numbers (*n*) and percentages. A value of—indicates a reported result of 0 (0). A blacked-out section indicates that there were no responses to the question/no data exists.

* For the purposes of this analysis, biomedical engineers/technicians (*n* = 4) were classified as non-patient care. MI indicates medical instruments.

**Table 4.** Glove use during cleaning product use by combined occupation categories and selected occupations.

Occupation (N)	Glove Use by Products N (%)							
	Detergents	Glass Cleaner	Quaternary Ammonium Compounds (Surface)		Alcohol (Surface)	Alcohol (MI)	Bleach (Surface)	Bleach (MI)
Total (559)	40 (57)	20 (47)	136 (87)		91 (64)	16 (80)	85 (85)	13 (87)
Admin (123)	2 (33)	1 (11)	9 (56)		8 (67)	1 (100)	3 (43)	
Non-patient care * (84)	18 (75)	9 (75)	26 (93)		22 (79)	5 (71)	31 (97)	6 (86)
Central Supply or Environmental Service Worker (10)	6 (100)	6 (100)	8 (100)		-	1 (50)	8 (100)	2 (100)
Lab Technician (30)	6 (75)	1 (100)	15 (100)		17 (94)	2 (100)	18 (100)	2 (100)
Maintenance (26)	5 (63)	2 (50)	3 (75)		3 (60)	-	3 (75)	-
Patient care (341)	20 (50)	10 (45)	101 (90)		61 (59)	10 (83)	51 (84)	7 (88)
Licensed Practical Nurse (18)		-	3 (75)		1 (25)		1 (50)	
Nurse (149)	14 (58)	7 (58)	65 (93)		34 (60)	6 (86)	33 (87)	3 (75)
Nursing Assistant (16)	2 (67)	1 (50%)	2 (100)		6 (100)	3 (100)	3 (100)	2 (100)
Other Nurse (57)	3 (38)	1 (33)	11 (85)		5 (56)	1 (100)	8 (73)	1 (100)
Other Technician (93)	1 (20)	1 (25)	17 (85)		13 (54)		5 (83)	
Other (11)								

Percentage of workers in each occupation or category who wear gloves when working with a specific chemical, expressed as a percentage of total question respondents in that occupation or category. Frequency of affirmative responses expressed using numbers (*n*) and percentages. A value of—indicates a reported result of 0 (0). A blacked-out section indicates that there were no responses to the question/no data exists.

* For the purposes of this analysis, biomedical engineers/technicians (*n* = 4) were classified as non-patient care. MI indicates medical instruments.

**Table 5.** Univariate models of glove use against participant characteristics/hand hygiene practices.

Effect	Category	Odds Ratio (95% CI)	Pr > Ltl
Occupation Category *	Non-Patient Care	21.3 (3.6, 125.9)	<0.05
	Patient Care	8.2 (1.8, 36.8)	<0.05
	Administration (Reference)	N/A	N/A
Product	Bleach	9.0 (2.6, 31.9)	<0.05
	Detergent	0.4 (0.1, 1.3)	0.12
	Quaternary Ammonium Compounds	13.4 (3.9, 46.4)	<0.05
	Glass Cleaner	0.3 (0.1, 1.3)	0.11
	Alcohol (Reference)	N/A	N/A
Days per Week Used Product	2–3	2.5 (1.1, 5.7)	<0.05
	4–5	3.9 (1.9, 8.2)	<0.05
	6–7	1.4 (0.4, 5.0)	0.61
	<=1 day/week (Reference)	N/A	N/A
Sex	Male	2.4 (0.7, 7.9)	0.15
	Female (Reference)	N/A	N/A
Race	Other	3.1 (0.6, 14.7)	0.16
	White (Reference)		
Handwashing Frequency	>10 times daily	2.0 (0.8, 5.0)	0.13
	<=10 times daily (Reference)	N/A	N/A
Hand Sanitizing Frequency	1–3 times daily	0.9 (0.2, 4.3)	0.87
	4–10 times daily	4.5 (0.9, 23.1)	0.07
	>10 times daily	4.3 (1.0, 19.0)	0.06
	<1 time daily (Reference)	N/A	N/A
Age		1.0 (0.9, 1.0)	0.07
Total Years Worked in Healthcare		1.0 (1.0, 1.0)	0.68
Total Hours Worked Per Week		1.0 (0.9, 1.1)	0.96
Skin Condition/Allergy	Atopic Dermatitis	1.1 (0.4, 3.3)	0.81
	Hives	0.6 (0.2, 1.8)	0.41
	Rash (work)	1.2 (0.3, 4.8)	0.81
	Rubber Allergy	7.3 (0.3, 154.2)	0.2

Results of a bivariate analysis of participant characteristics associated with glove use. Statistical significance *p* < 0.05. Reference groups for each effect were as follows: Product (alcohol), Days/Week Used Product (<=1 day/week), Sex (Female), Race (White), Occupation Category (Admin), Handwashing Frequency (<=10 times daily), Hand Sanitizing Frequency (<1 time daily).

* For the purposes of this analysis, biomedical engineers/technicians (*n* = 4) were classified as non-patient care.

**Table 6.** Skin disorders and rubber allergy distribution by participant characteristics, N (%).

Overall		Atopic Dermatitis *n* = 116	Hives *n* = 105	Rash (Work) *n* = 34	Rubber Allergy *n* = 11
Occupation	Administration	28 (24.1)	27 (25.7)	5 (14.7)	5 (45.5)
	Central Supply or Environmental Service Worker	-	-	-	-
	Lab Technician	6 (5.2)	7 (6.7)	-	-
	Maintenance	4 (3.4)	5 (4.8)	-	-
	Pharmacist or Pharmacy Technician	-	-	-	-
	Other	-	3 (2.9%)	-	-
	Licensed Practical Nurse	6 (5.2)	-	-	-
	Nurse	30 (25.9)	28 (26.7)	15 (44.1)	3 (27.3)
	Nursing Assistant	8 (6.9)	4 (3.8)	-	-
	Other Nurse	17 (14.7)	10 (9.5)	3 (8.8)	-
	Other Technician	13 (11.2)	16 (15.2)	4 (11.8)	-
	Respiratory Therapist or Technician	-	-	-	-
Sex	Male	21 (18)	15 (14)	6 (18)	-
	Female	95 (82)	90 (86)	28 (82)	11 (100)
Race	White	102 (88)	93 (89)	31 (91)	8 (73)
	Other	14 (12)	12 (11)	3 (9)	3 (27)
Age	20–39	32 (28)	37 (35)	14 (41)	-
	40–49	30 (26)	23 (22)	8 (24)	5 (45)
	50+	54 (46)	45(43)	12 (35)	4 (36)
Total Years Worked in Healthcare	0–9	37 (32)	35 (33)	9 (26)	3 (27)
	10–29	45 (39)	49 (47)	18 (53)	7 (64)
	30+	34 (30)	21 (20)	7 (21)	-
Hours Worked Per Week	<40	17 (15)	11 (10)	3 (9)	-
	40+	98 (85)	94 (90)	31 (91)	9 (82)
Handwashing Frequency	<=10 Times Per Day	66 (58.9)	57 (57)	11 (33.3)	5 (45)
	>10 Times Per Day	46 (41.1)	43 (43)	22 (66.7)	6 (55)
Hand Sanitizer Use	<=10 Times Per Day *	65 (56)	60 (57.1)	14 (41.2)	5 (56)
	>10 Times Per Day	40 (34.5)	36 (34.3)	17 (50)	4 (44)
Current Asthma	Yes	33 (28)	30 (29)	8 (24)	5 (45)
	No	83 (72)	75 (71)	26 (76)	6 (55)
Allergic status ^+^	Positive	116 (100)	82 (78)	28 (82)	11 (100)
	Negative	-	23 (22)	6 (18)	-

Distribution of skin symptoms by sex, race, age, occupation, total years worked in healthcare, hours worked per week, product use, hand sanitizer use, and handwashing frequency. Participants were considered as positive for hives/rash if an outbreak occurred in the past 12 months. Participants were considered as positive for allergic reactions and dermatitis if an instance had ever occurred within the participant’s lifetime. A value of—indicates a reported result of two or fewer workers experiencing the symptom

* Excludes “Never Use” hand sanitizer.

+ Positive status defined as ever having atopic dermatitis, allergic eyes symptoms, or seasonal allergic rhinitis.

## Data Availability

The data are not publicly available due to privacy concerns regarding personally identifiable information of the survey respondents.
