# Supplementary material for: Characterization of Cleaning and Disinfection Product Use, Glove Use, and Skin Disorders by Healthcare Occupations in a Midwestern Healthcare Facility
Source: Buildings (Basel). Author manuscript; Available in PMC 2024 Apr 22. (PMC11034745; doi:10.3390/buildings12122216)
Supplement: Supplementary Materials [file NIHMS1958359-supplement-Supplementary_Materials.pdf]

Race
- ☐ White
- ☐ African-American or Black
- ☐ Asian
- ☐ American Indian or Alaska Native
- ☐ Native Hawaiian or Other Pacific Islander
- ☐ Other-please specify

Other Race ______________________________

SECTION II: HEALTH INFORMATION

BREATHING PROBLEMS

Have you had wheezing or whistling in your chest at any time

in the last 12 months?
- ☐ Yes
- ☐ No

in the last 4 weeks?
- ☐ Yes
- ☐ No

Have you woken up with a feeling of tightness in your chest first thing in the morning at any time

in the last 12 months?
- ☐ Yes
- ☐ No

in the last 4 weeks?
- ☐ Yes
- ☐ No

Have you at any time had an attack of shortness of breath that came on during the day when you were not doing anything strenuous

in the last 12 months?
- ☐ Yes
- ☐ No

in the last 4 weeks?
- ☐ Yes
- ☐ No

Have you had an attack of shortness of breath that came on after you stopped exercising at any time

in the last 12 months?
- ☐ Yes
- ☐ No

in the last 4 weeks?
- ☐ Yes
- ☐ No

Have you at any time been woken up at night by an attack of shortness of breath

in the last 12 months?
- ☐ Yes
- ☐ No

in the last 4 weeks?
- ☐ Yes
- ☐ No

Have you had an attack of asthma at any time

in the last 12 months?
- ☐ Yes
- ☐ No

in the last 4 weeks?
- ☐ Yes
- ☐ No

Are you currently taking any medications (including inhalers, aerosols, or tablets) for asthma?
- ☐ Yes
- ☐ No

How old were you when you first had any of these types of breathing problems?

____
(in years)

Which one of the following statements best describes your breathing problems when you are on vacation, on your day off, or on a lay-off?

☐ Breathing problems are better away from work.
☐ Breathing problems are worse away from work.
☐ Breathing problems are no different away from work.

Were these breathing problems ever so severe that you had to take time off work?

☐ Yes
☐ No

In the past 12 months, about how many days did you take off of work because of your breathing?

____
(number of days)

ADDITIONAL RESPIRATORY INFORMATION

COUGH

Do you usually cough initially after waking up, or first thing in the morning? (Count a cough with first smoke or on first going out-of-doors. Exclude clearing the throat.)

☐ Yes
☐ No

Do you usually cough at all during the rest of the day or at night?

☐ Yes
☐ No

Do you cough like this on most days for at least 3 months each year?

☐ Yes
☐ No

How many years have you had this cough?

____
(years)

PHLEGM

Do you usually bring up phlegm at all upon getting up, or first thing in the morning? (Count phlegm with first smoke or on first going out-of-doors. Exclude phlegm from the nose. Count swallowed phlegm.)

☐ Yes
☐ No

Do you usually bring up phlegm at all during the rest of the day or at night?

☐ Yes
☐ No

Do you bring up phlegm like this on most days for at least 3 months each year?

☐ Yes
☐ No

For how many years have you had phlegm like this?

____
(years)

Which ONE of the following statements best describes your breathing?

☐ I never or only rarely have trouble with my breathing.
☐ I have regular trouble with my breathing, but it always gets completely better.
☐ My breathing is never quite right.

When you are in a dusty part of the house or with animals (for instance dogs, cats, or horses) or near feathers (including down pillows, down quilts, or down comforters) do you ever:

Get a feeling of tightness in your chest?

☐ Yes
☐ No

Start to feel short of breath?

☐ Yes
☐ No

ASTHMA

Have you ever had asthma?

☐ Yes
☐ No

How old were you when your asthma started? ______________________ (years old)

Was your asthma confirmed/diagnosed by a doctor or a healthcare professional?
☐ Yes
☐ No

Do you still have asthma?
☐ Yes
☐ No

How old were you when your asthma stopped? ______________________ (years old)

Have you had a "cold" or upper respiratory infection in the last four weeks?
☐ Yes
☐ No

EYES, NOSE, SINUS SYMPTOMS

On most days, do you have a stuffy nose or drainage from the back of your nose into your throat?
☐ Yes
☐ No

Have you had 5 or more attacks of sneezing or blocked, itchy, or runny nose? (Count attacks of sneezing 4 or more times in a row. Count attacks of blocked, itchy, or runny nose lasting 15 minutes or more. Do not count episodes of sneezing less than 4 times in a row or blocked, itchy, or runny nose lasting less than 15 minutes.)

in the last 12 months?
☐ Yes
☐ No

in the last 4 weeks?
☐ Yes
☐ No

Have you had 5 or more attacks of red, itchy, or watery eyes? (Count attacks of red, itchy, watery eyes lasting 15 minutes or more. Do not count episodes lasting less than 15 minutes.)

in the last 12 months?
☐ Yes
☐ No

in the last 4 weeks?
☐ Yes
☐ No

How old were you when you first noticed these nose or eye symptoms? ______________________ (years old)

Which ONE of the following statements best describes your nose or eye symptoms when you are on vacation, on weekends, or on a day-off?
☐ Nose or eye symptoms are better away from work.
☐ Nose or eye symptoms are worse away from work.
☐ Nose or eye symptoms are no different away from work.

SKIN

During the past 12 months, have you had hives? (Hives are raised swellings on the skin that itch alot and look like mosquito bites. Surrounding skin may be red. Other names for hives are wheals or urticaria.)
☐ Yes
☐ No

When AT WORK during the past 12 months, have gloves (latex, rubber, or nitrile) caused you to have a rash on your hands other than hives that was red, cracked, peeling, bumpy, blistered, or oozing and possibly itchy? (Names for this rash might include dermatitis or eczema.)
☐ Yes
☐ No

When AT HOME during the past 12 months, have gloves (latex, rubber, or nitrile) caused you to have a rash on your hands other than hives that was red, cracked, peeling, bumpy, blistered, or oozing and possibly itchy? (Names for this rash might include dermatitis or eczema.)
☐ Yes
☐ No

SEVERE ALLERGIC REACTION

Have you EVER had a life-threatening allergic reaction with ANY of the following: low blood pressure; swelling of lips, tongue, or throat; difficulty breathing; or loss of consciousness? (The name for this kind of allergic reaction is anaphylaxis).

☐ Yes
☐ No

When was your last severe allergic reaction?

____
(month-2 digits/year-4 digits (e.g. 06/2006))

Which ONE of the following do you think caused this severe allergic reaction?

☐ Insect sting
☐ Medication
☐ Food
☐ Latex glove or other latex device
☐ Radiocontrast dye (dye for x-ray test)
☐ Other (specify)

Other-specify

____

Have ANY of the items listed below EVER caused you to have ANY of these allergic reactions: swelling of lips, tongue, or throat; wheezing, chest tightness, shortness of breath, or asthma; allergic reactions in eyes, nose or sinuses; hives; or a life threatening reaction (anaphylaxis)?

Objects made of natural rubber. Examples include: household rubber gloves, dental dams, balloons, rubber bands, rubber balls including "Koosh" balls, rubber handles, baby bottle nipples, and/or teething rings, condoms, diaphragms?

☐ Yes
☐ No

Any of the following foods: Banana, avocado, chestnut, kiwi, potato, tomato?

☐ Yes
☐ No

Dental or obstetric-gynecologic (OB-GYN) exams?

☐ Yes
☐ No

PAST MEDICAL HISTORY

Have you ever had the following medical problems (please give an answer for each):

Atopic Dermatitis (Eczema)

☐ Yes
☐ No

Allergic Eye Symptoms

☐ Yes
☐ No

Sinusitis (confirmed by a physician)

☐ Yes
☐ No

Seasonal Allergic Rhinitis (Hayfever, seasonal nasal allergies)

☐ Yes
☐ No

Other types of nasal allergies

☐ Yes
☐ No

Drug Allergies

☐ Yes
☐ No

Chronic Bronchitis

☐ Yes
☐ No

Emphysema

☐ Yes
☐ No

Cardiac (heart) Disease

☐ Yes
☐ No

FAMILY HISTORY

Have any close relatives (parents, brothers, sisters, or children) ever had asthma?
- ☐ No
- ☐ Yes
- ☐ Don't know

Please check ALL close relatives that have had asthma:
- ☐ Mother or Father
- ☐ Sister or Brother
- ☐ Daughter or Son

Have any close relatives (parents, brothers, sisters, or children) ever had eczema (atopic dermatitis) or nasal allergies (hay fever)?
- ☐ No
- ☐ Yes
- ☐ Don't know

Please check ALL that have had eczema (atopic dermatitis) or nasal allergies (hay fever):
- ☐ Mother or Father
- ☐ Sister or Brother
- ☐ Daughter or Son

SMOKING

Have you EVER smoked cigarettes regularly? (Please mark "No" if you have smoked less than 100 cigarettes in your lifetime.)
- ☐ Yes
- ☐ No

How old were you when you first started smoking cigarettes regularly?
____
(years old)

Do you still smoke cigarettes?
- ☐ Yes
- ☐ No

How old were you when you last gave up smoking?
____
(years old)

Over the years that you smoked, on the average, approximately how many cigarettes per day did you smoke?
____
(cigarettes per day)

Do you currently smoke a pipe or cigar?
- ☐ Yes
- ☐ No

SECTION III: OCCUPATIONAL INFORMATION

The following questions will ask about your work in health care and your current job at this medical center.

How many TOTAL years have you worked in health care, including your time in training?
____
(years)

When did you begin working at the Milwaukee VA?
____
(month-2 digits/year-4 digits (e.g. 06/2006))

When did you begin your CURRENT job at the Milwaukee VA?
____
(month-2 digits/year-4 digits (e.g. 06/2006))

What department are you currently assigned to?

- ☐ Medical Center Director
- ☐ Chief of Staff
- ☐ Anesthesia Division
- ☐ Clinical Support Division
- ☐ Facility Management Division
- ☐ Great Lakes Finance Service
- ☐ Great Lakes Acquisition Center
- ☐ Human Resources
- ☐ Medical Imaging
- ☐ Medical Information Systems
- ☐ Medical Subspecialties Division
- ☐ Mental Health
- ☐ Nursing, Med/Surg and Critical Care
- ☐ Pathology and Laboratory Medicine Service
- ☐ Primary Care
- ☐ Prosthetics
- ☐ Rehabilitation, Extended and Community Care
- ☐ Research
- ☐ Spinal Cord Injury
- ☐ Surgical Subspecialites
- ☐ Utilization Management
- ☐ Other

Other department - specify ________________________________

Which ONE of the following best describes your CURRENT job?

- ☐ Administrator
- ☐ Audiologist
- ☐ Ambulatory Surgical Care
- ☐ Anesthesiology Tech.
- ☐ Biomedical Engineer
- ☐ Biomedical Tech.
- ☐ Cardiovascular Tech.
- ☐ Central Supply Tech (SPD)
- ☐ Clinical Laboratory worker (Medical Tech/Pathology/Histology/Cytology)
- ☐ Dental Assistant
- ☐ Dental Hygienist
- ☐ Dental Lab Technician
- ☐ Dentist
- ☐ Dialysis Technician
- ☐ Dietician
- ☐ Endoscopy Technician
- ☐ ENT
- ☐ Environmental Health & Safety
- ☐ Facility Maintenance Worker
- ☐ Food Service Worker
- ☐ Healthcare/Medical Tech.
- ☐ Housekeeper (EMS)
- ☐ Lab Technician
- ☐ Material Handler/Shipping
- ☐ Medical Equipment Preparer
- ☐ Nurse - Anesthetist
- ☐ Nurse - Operating Room
- ☐ Nurse - Clinical (not Operating Room)
- ☐ Nurse Practitioner
- ☐ Nursing Assistant
- ☐ Nuclear Med. Tech.
- ☐ Occupational Therapist
- ☐ Office/Medical Records Worker
- ☐ Optometrist
- ☐ Orthotist & Prosthetist
- ☐ Pathologist
- ☐ Patient Care Asst. (Transport, etc)
- ☐ Pharmacist
- ☐ Pharmacist Tech.
- ☐ Phlebotomist
- ☐ Physician Assistant
- ☐ Physician - Anesthesiology
- ☐ Physician - Nonsurgical
- ☐ Physician - Surgical
- ☐ Physical Therapist
- ☐ Psychologist
- ☐ Podiatrist
- ☐ Prosthetics Lab
- ☐ Radiology File Clerk
- ☐ Radiology Technician
- ☐ Research Laboratory Worker
- ☐ Respiratory Therapist
- ☐ Social Worker
- ☐ Speech Therapist
- ☐ Surgical/OR Technician
- ☐ Ward Clerk
- ☐ Other

Other job-specify ________________________

Please specify your registration or certification in your occupation/discipline (e.g. RN, CNA, CST, etc). ________________________

How many DAYS PER WEEK do you usually work at this medical center? ________________________
(days per week (e.g. 3.0 or 5.5))

How many TOTAL HOURS per WEEK do you usually work at this medical center? __________________ (hours per week (e.g. 8.0 or 24.5 or 46.0))

During a USUAL WORK WEEK at this medical center, please fill in the number of hours you work PER WEEK in each of the following areas. (In areas that you do not work, leave blank.):

Patient Care Areas

Surgery: Operating Room __________________ (number of hours (e.g. 3.0 or 5.5))

Surgery: Recovery Room __________________ (number of hours (e.g. 3.0 or 5.5))

Outpatient Surgical Areas (minor procedures, surgical clinic) __________________ (number of hours (e.g. 3.0 or 5.5))

Dental Clinic __________________ (number of hours (e.g. 3.0 or 5.5))

Hemodialysis __________________ (number of hours (e.g. 3.0 or 5.5))

Women's Health Clinic __________________ (number of hours (e.g. 3.0 or 5.5))

Endoscopy Unit (Cystoscopy, GI, Bronchoscopy) __________________ (number of hours (e.g. 3.0 or 5.5))

Cardiac Catheterization Lab __________________ (number of hours (e.g. 3.0 or 5.5))

Noninvasive Cardiac Lab __________________ (number of hours (e.g. 3.0 or 5.5))

Pulmonary Function Lab __________________ (number of hours (e.g. 3.0 or 5.5))

Critical Care Unit (CCU, MICU, SICU, etc) __________________ (number of hours (e.g. 3.0 or 5.5))

Medical/Surgical Ward (inpatient wards other than psychiatric) __________________ (number of hours (e.g. 3.0 or 5.5))

Medical Outpatient Clinic (nonsurgical outpatient) __________________ (number of hours (e.g. 3.0 or 5.5))

Psychiatric or Substance Abuse Ward or Clinic __________________ (number of hours (e.g. 3.0 or 5.5))

Radiology/Nuclear Medicine __________________ (number of hours (e.g. 3.0 or 5.5))

Audiology __________________ (number of hours (e.g. 3.0 or 5.5))

Emergency Room __________________ (number of hours (e.g. 3.0 or 5.5))

Admissions __________________ (number of hours (e.g. 3.0 or 5.5))

Phlebotomy (blood drawing area) __________________ (number of hours (e.g. 3.0 or 5.5))

Please specify other patient care area not listed above. __________________

Other Patient Care Area (Specify time) ________________________________
(number of hours (e.g. 3.0 or 5.5))

Non-Patient Care Areas

Clinical laboratory ________________________________
(number of hours (e.g. 3.0 or 5.5))

Dental Laboratory ________________________________
(number of hours (e.g. 3.0 or 5.5))

Prosthetics Laboratory ________________________________
(number of hours (e.g. 3.0 or 5.5))

Pharmacy ________________________________
(number of hours (e.g. 3.0 or 5.5))

Cafeteria/Kitchen ________________________________
(number of hours (e.g. 3.0 or 5.5))

Office/Medical Records ________________________________
(number of hours (e.g. 3.0 or 5.5))

Maintenance/Engineering ________________________________
(number of hours (e.g. 3.0 or 5.5))

Supply Room/Warehouse ________________________________
(number of hours (e.g. 3.0 or 5.5))

Central Supply and Distribution ________________________________
(number of hours (e.g. 3.0 or 5.5))

Research Laboratory ________________________________
(number of hours (e.g. 3.0 or 5.5))

Please specify other non-patient care area not listed above. ________________________________

Other non-Patient Care Area (Specify time) ________________________________
(number of hours (e.g. 3.0 or 5.5))

Hand Washing and Use of Hand Sanitizers in the Workplace (Current Job)

How many times per day do you wash your hands?
- ☐ Never
- ☐ less than 1 time per day
- ☐ 1-3 times per day
- ☐ 4-10 times per day
- ☐ greater than 10 times per day

How many times per day do you disinfect your hands using liquid hand sanitizer?
- ☐ Never
- ☐ less than 1 time per day
- ☐ 1-3 times per day
- ☐ 4-10 times per day
- ☐ greater than 10 times per day

SECTION IV: EXPOSURES AT WORK

Exposure to Mold and Moisture in the Workplace (Current Job)

Do you have a primary work station where you spend more than 50% of your work time?
- ☐ Yes
- ☐ No

What building is your primary work station located in?
- ☐ Bldg # 4
- ☐ Bldg # 5
- ☐ Bldg # 6
- ☐ Bldg # 7
- ☐ Bldg # 43
- ☐ Bldg # 70
- ☐ Bldg # 109
- ☐ Bldg # 111
- ☐ Bldg # 123
- ☐ Bldg # 144

Where is your primary work station located (floor/ward/wing)? ______________________ (floor/ward/wing)

Have you observed any of the following in your primary work station (floor/ward/wing) in the past?

Water leakage, wet spots or water damage indoors on walls, floors, or ceilings?

in the past 2 years? ☐ Yes ☐ No

in the past 6 months? ☐ Yes ☐ No

in the past 4 weeks? ☐ Yes ☐ No

Condensation (fog) on the inside surfaces of windows or on cold inside surfaces?

in the past 2 years? ☐ Yes ☐ No

in the past 6 months? ☐ Yes ☐ No

in the past 4 weeks? ☐ Yes ☐ No

Wet carpet from leaks, flooding, or frequent spills?

in the past 2 years? ☐ Yes ☐ No

in the past 6 months? ☐ Yes ☐ No

in the past 4 weeks? ☐ Yes ☐ No

Visible mold growth (not food) indoors on walls, floors, or ceilings?

in the past 2 years? ☐ Yes ☐ No

in the past 6 months? ☐ Yes ☐ No

in the past 4 weeks? ☐ Yes ☐ No

Odor of mold, mildew or musty smell (not from food)?

in the past 2 years? ☐ Yes ☐ No

in the past 6 months? ☐ Yes ☐ No

in the past 4 weeks?
- ☐ Yes
- ☐ No

Exposure to Construction Material in the Workplace (Current Job)

Have there been renovations or construction at your primary work station (floor/ward/wing)?

in the past 2 years?
- ☐ Yes
- ☐ No

in the past 6 months?
- ☐ Yes
- ☐ No

in the past 4 weeks?
- ☐ Yes
- ☐ No

Was the renovation related to water damage or mold?
- ☐ Yes
- ☐ No

Painting walls or fixtures?

in the past 2 years?
- ☐ Yes
- ☐ No

in the past 6 months?
- ☐ Yes
- ☐ No

in the past 4 weeks?
- ☐ Yes
- ☐ No

Ripping out and replacing walls, woodwork, or partitions?

in the past 2 years?
- ☐ Yes
- ☐ No

in the past 6 months?
- ☐ Yes
- ☐ No

in the past 4 weeks?
- ☐ Yes
- ☐ No

Ripping out and replacing carpets or fixed furniture?

in the past 2 years?
- ☐ Yes
- ☐ No

in the past 6 months?
- ☐ Yes
- ☐ No

in the past 4 weeks?
- ☐ Yes
- ☐ No

Exposure to Sterilizing & High-Level Disinfecting Products Used on Medical Instuments (Current Job)

Do you sterilize or high-level disinfect medical instruments, including dental instruments or ventilator parts, in central supply or other locations such as endoscopy and bronchoscopy units, hemodialysis units, operating rooms or other clinical settings?
- ☐ Yes
- ☐ No

Acetic Acid - do you use this chemical or product?
- ☐ No
- ☐ Yes
- ☐ Don't know

Acetic Acid - used how many days per week?	☐ less than 1<br>☐ 1<br>☐ 2<br>☐ 3<br>☐ 4<br>☐ 5<br>☐ 6<br>☐ 7
Acetic Acid - are gloves worn with this chemical or product?	☐ none<br>☐ Nitrile<br>☐ Latex or Vinyl<br>☐ Don't Know
Alcohol - do you use this chemical or product?	☐ No<br>☐ Yes<br>☐ Don't know
Alcohol - used how many days per week?	☐ less than 1<br>☐ 1<br>☐ 2<br>☐ 3<br>☐ 4<br>☐ 5<br>☐ 6<br>☐ 7
Alcohol - are gloves worn with this chemical or product?	☐ none<br>☐ Nitrile<br>☐ Latex or Vinyl<br>☐ Don't Know
Bleach or chlorine such as Chlorox* - do you use this chemical or product?	☐ No<br>☐ Yes<br>☐ Don't know
Bleach or chlorine such as Chlorox* - used how many days per week?	☐ less than 1<br>☐ 1<br>☐ 2<br>☐ 3<br>☐ 4<br>☐ 5<br>☐ 6<br>☐ 7
Bleach or chlorine such as Chlorox* - are gloves worn with this chemical or product?	☐ none<br>☐ Nitrile<br>☐ Latex or Vinyl<br>☐ Don't Know
Enzymatic cleaners, such as Asepti-Zyme*, 3M Rapid Multi-Enzyme* - do you use this chemical or product?	☐ No<br>☐ Yes<br>☐ Don't know
Enzymatic cleaners, such as Asepti-Zyme*, 3M Rapid Multi-Enzyme* - used how many days per week?	☐ less than 1<br>☐ 1<br>☐ 2<br>☐ 3<br>☐ 4<br>☐ 5<br>☐ 6<br>☐ 7
Enzymatic cleaners, such as Asepti-Zyme*, 3M Rapid Multi-Enzyme* - are gloves worn with this chemical or product?	☐ none<br>☐ Nitrile<br>☐ Latex or Vinyl<br>☐ Don't Know

Ethylene oxide in compressed-gas cylinders or single-dose cartridges - do you use this chemical or product?

☐ No
☐ Yes
☐ Don't know

Ethylene oxide in compressed-gas cylinders or single-dose cartridges - used how many days per week?

☐ less than 1
☐ 1
☐ 2
☐ 3
☐ 4
☐ 5
☐ 6
☐ 7

Ethylene oxide in compressed-gas cylinders or single-dose cartridges - are gloves worn with this chemical or product?

☐ none
☐ Nitrile
☐ Latex or Vinyl
☐ Don't Know

Ethylene oxide in glass ampules - do you use this chemical or product?

☐ No
☐ Yes
☐ Don't know

Ethylene oxide in glass ampules - used how many days per week?

☐ less than 1
☐ 1
☐ 2
☐ 3
☐ 4
☐ 5
☐ 6
☐ 7

Ethylene oxide in glass ampules - are gloves worn with this chemical or product?

☐ none
☐ Nitrile
☐ Latex or Vinyl
☐ Don't Know

Formaldehyde - do you use this chemical or product?

☐ No
☐ Yes
☐ Don't know

Formaldehyde - used how many days per week?

☐ less than 1
☐ 1
☐ 2
☐ 3
☐ 4
☐ 5
☐ 6
☐ 7

Formaldehyde - are gloves worn with this chemical or product?

☐ none
☐ Nitrile
☐ Latex or Vinyl
☐ Don't Know

Glutaraldehydes such as Cidex*, Metricide*, Rapicide*, Wavicide* - do you use this chemical or product?

☐ No
☐ Yes
☐ Don't know

Glutaraldehydes such as Cidex*, Metricide*, Rapicide*, Wavicide* - used how many days per week?

☐ less than 1
☐ 1
☐ 2
☐ 3
☐ 4
☐ 5
☐ 6
☐ 7

Glutaraldehydes such as Cidex*, Metricide*, Rapicide*, Wavicide*- are gloves worn with this chemical or product?	☐ none<br>☐ Nitrile<br>☐ Latex or Vinyl<br>☐ Don't Know
Glutaraldehyde and isopropanol combinations such as Aldahol III* - do you use this chemical or product?	☐ No<br>☐ Yes<br>☐ Don't know
Glutaraldehyde and isopropanol combinations such as Aldahol III* - used how many days per week?	☐ less than 1<br>☐ 1<br>☐ 2<br>☐ 3<br>☐ 4<br>☐ 5<br>☐ 6<br>☐ 7
Glutaraldehyde and isopropanol combinations such as Aldahol III* - are gloves worn with this chemical or product?	☐ none<br>☐ Nitrile<br>☐ Latex or Vinyl<br>☐ Don't Know
Glutaraldehyde and phenol/phenate combinations such as Sporicidin* - do you use this chemical or product?	☐ No<br>☐ Yes<br>☐ Don't know
Glutaraldehyde and phenol/phenate combinations such as Sporicidin* - used how many days per week?	☐ less than 1<br>☐ 1<br>☐ 2<br>☐ 3<br>☐ 4<br>☐ 5<br>☐ 6<br>☐ 7
Glutaraldehyde and phenol/phenate combinations such as Sporicidin* - are gloves worn with this chemical or product?	☐ none<br>☐ Nitrile<br>☐ Latex or Vinyl<br>☐ Don't Know
Hydrogen peroxides such as Accell*, Optim*, Resert XL*, Sporox* - do you use this chemical or product?	☐ No<br>☐ Yes<br>☐ Don't know
Hydrogen peroxides such as Accell*, Optim*, Resert XL*, Sporox* - used how many days per week?	☐ less than 1<br>☐ 1<br>☐ 2<br>☐ 3<br>☐ 4<br>☐ 5<br>☐ 6<br>☐ 7
Hydrogen peroxides such as Accell*, Optim*, Resert XL*, Sporox* - are gloves worn with this chemical or product?	☐ none<br>☐ Nitrile<br>☐ Latex or Vinyl<br>☐ Don't Know
Hydrogen peroxide and peracetic acid combinations such as Acecide*, EndoSpor Plus*, Metrex*, Peract* - do you use this chemical or product?	☐ No<br>☐ Yes<br>☐ Don't know

Hydrogen peroxide and peracetic acid combinations such as Acecide*, EndoSpor Plus*, Metrex*, Peract*- used how many days per week?

- [ ] less than 1
- [ ] 1
- [ ] 2
- [ ] 3
- [ ] 4
- [ ] 5
- [ ] 6
- [ ] 7

Hydrogen peroxide and peracetic acid combinations such as Acecide*, EndoSpor Plus*, Metrex*, Peract* - are gloves worn with this chemical or product?

- [ ] none
- [ ] Nitrile
- [ ] Latex or Vinyl
- [ ] Don't Know

Hydrogen peroxide gas plasma such as Sterad* - do you use this chemical or product?

- [ ] No
- [ ] Yes
- [ ] Don't know

Hydrogen peroxide gas plasma such as Sterad*- used how many days per week?

- [ ] less than 1
- [ ] 1
- [ ] 2
- [ ] 3
- [ ] 4
- [ ] 5
- [ ] 6
- [ ] 7

Hydrogen peroxide gas plasma such as Sterad* - are gloves worn with this chemical or product?

- [ ] none
- [ ] Nitrile
- [ ] Latex or Vinyl
- [ ] Don't Know

Hypochlorite or Hypochlorous acids such as Sterilox* - do you use this chemical or product?

- [ ] No
- [ ] Yes
- [ ] Don't know

Hypochlorite or Hypochlorous acids such as Sterilox*- used how many days per week?

- [ ] less than 1
- [ ] 1
- [ ] 2
- [ ] 3
- [ ] 4
- [ ] 5
- [ ] 6
- [ ] 7

Hypochlorite or Hypochlorous acids such as Sterilox* - are gloves worn with this chemical or product?

- [ ] none
- [ ] Nitrile
- [ ] Latex or Vinyl
- [ ] Don't Know

Ortho-phthalaldehydes such as Cidex OPA*, Opaciden* - do you use this chemical or product?

- [ ] No
- [ ] Yes
- [ ] Don't know

Ortho-phthalaldehydes such as Cidex OPA*, Opaciden*- used how many days per week?

- [ ] less than 1
- [ ] 1
- [ ] 2
- [ ] 3
- [ ] 4
- [ ] 5
- [ ] 6
- [ ] 7

Ortho-phthalaldehydes such as Cidex OPA*, Opaciden* - are gloves worn with this chemical or product?

- [ ] none
- [ ] Nitrile
- [ ] Latex or Vinyl
- [ ] Don't Know

Peracetic acid such as Steris* - do you use this chemical or product?

- ☐ No
- ☐ Yes
- ☐ Don't know

Peracetic acid such as Steris*- used how many days per week?

- ☐ less than 1
- ☐ 1
- ☐ 2
- ☐ 3
- ☐ 4
- ☐ 5
- ☐ 6
- ☐ 7

Peracetic acid such as Steris* - are gloves worn with this chemical or product?

- ☐ none
- ☐ Nitrile
- ☐ Latex or Vinyl
- ☐ Don't Know

Please write any other sterilizing or high-level disinfecting chemicals or product names you use on medical instruments.

Name brand or product # 1 ______________________________

Above named product- used how many days per week?

- ☐ less than 1
- ☐ 1
- ☐ 2
- ☐ 3
- ☐ 4
- ☐ 5
- ☐ 6
- ☐ 7

Above named product- are gloves worn with this?

- ☐ none
- ☐ Nitrile
- ☐ Latex or Vinyl
- ☐ Don't Know

Name brand or product # 2 ______________________________

Above named product- used how many days per week?

- ☐ less than 1
- ☐ 1
- ☐ 2
- ☐ 3
- ☐ 4
- ☐ 5
- ☐ 6
- ☐ 7

Above named product- are gloves worn with this?

- ☐ none
- ☐ Nitrile
- ☐ Latex or Vinyl
- ☐ Don't Know

Name brand or product # 3 ______________________________

Above named product- used how many days per week?

- ☐ less than 1
- ☐ 1
- ☐ 2
- ☐ 3
- ☐ 4
- ☐ 5
- ☐ 6
- ☐ 7

Above named product- are gloves worn with this?

- ☐ none
- ☐ Nitrile
- ☐ Latex or Vinyl
- ☐ Don't Know

Name brand or product # 4 __________________________________

Above named product- used how many days per week?
- ☐ less than 1
- ☐ 1
- ☐ 2
- ☐ 3
- ☐ 4
- ☐ 5
- ☐ 6
- ☐ 7

Above named product- are gloves worn with this?
- ☐ none
- ☐ Nitrile
- ☐ Latex or Vinyl
- ☐ Don't Know

Name brand or product # 5 __________________________________

Above named product- used how many days per week?
- ☐ less than 1
- ☐ 1
- ☐ 2
- ☐ 3
- ☐ 4
- ☐ 5
- ☐ 6
- ☐ 7

Above named product- are gloves worn with this?
- ☐ none
- ☐ Nitrile
- ☐ Latex or Vinyl
- ☐ Don't Know

Name brand or product # 6 __________________________________

Above named product- used how many days per week?
- ☐ less than 1
- ☐ 1
- ☐ 2
- ☐ 3
- ☐ 4
- ☐ 5
- ☐ 6
- ☐ 7

Above named product- are gloves worn with this?
- ☐ none
- ☐ Nitrile
- ☐ Latex or Vinyl
- ☐ Don't Know

On a typical day when you use sterilants or high-level disinfectants, how many TIMES PER DAY do you use these products?
- ☐ less than 1 time per day
- ☐ 1-3 times per day
- ☐ 4-10 times per day
- ☐ greater than10 times per day

On a typical day when you use sterilants or high-level disinfectants, how many HOURS PER DAY do you use these products?
- ☐ less than 1 hour
- ☐ 1-2 hours
- ☐ greater than 2-4 hours
- ☐ greater than 4-6 hours
- ☐ greater than 6 hours

Do you ever prepare medical instruments for sterilization by manually disassembling instruments, removing gross contaminants or flushing gross contaminents and waste?
- ☐ Yes
- ☐ No

When you remove gross contaminants and waste from scopes and instruments, please indicate how many days per week, times per week, duration of task and the type of gloves used when you perform this task.

Days per week ______________________ (number of days per week)

Times per day ______________________ (number of times per day)

Duration of task - please estimate the approximate number of minutes. ______________________ (number of minutes per task)

Type of gloves worn with task
- ☐ none
- ☐ Nitrile
- ☐ Latex or Vinyl
- ☐ Don't Know

Do you ever prepare medical instruments for sterilization by refilling or changing sterilization solutions?
- ☐ Yes
- ☐ No

When you refill or change sterilization solutions, please indicate how many days per week, times per week, duration of task and the type of gloves used when you perform this task.

Days per week ______________________ (number of days per week)

Times per day ______________________ (number of times per day)

Duration of task - please estimate the approximate number of minutes. ______________________ (number of minutes per task)

Type of gloves worn with task
- ☐ none
- ☐ Nitrile
- ☐ Latex or Vinyl
- ☐ Don't Know

Do you ever manually sterilize medical instruments?
- ☐ Yes
- ☐ No

When you manually sterilize medical instruments, please indicate how many days per week, times per week, duration of task and the type of gloves used when you perform this task.

Days per week ______________________ (number of days per week)

Times per day ______________________ (number of times per day)

Duration of task - please estimate the approximate number of minutes. ______________________ (number of minutes per task)

Type of gloves worn with task
- ☐ none
- ☐ Nitrile
- ☐ Latex or Vinyl
- ☐ Don't Know

Do you use a sterilant emersion container when you manually sterilize or high-level disinfect medical instruments?
- ☐ Yes
- ☐ No

When using a sterilants emersion container please select all conditions that apply.
- ☐ Enclosed box or exhaust hood used
- ☐ Emersion box equipped with a tight fitting lid
- ☐ Local exhaust ventilation is used in room
- ☐ None of the conditions apply

Do you ever sterilize medical instruments using automated endoscope reprocessing systems or other automated systems?
- ☐ Yes
- ☐ No

Do you perform the task of operating EtO sterilizer?
☐ No
☐ Yes
☐ Don't know

How many days per week do you operate EtO sterilizer?
____
(number of days per week)

How many times per day do you operate EtO sterilizer?
____
(number of times per day)

Duration of task - please estimate the approximate number of minutes to operate the EtO sterilizer each time.
____
(number of minutes per task)

Is system closed while operating EtO sterilizer?
☐ No
☐ Yes
☐ Don't know

Is local exhaust ventilation used while operating EtO sterilizer?
☐ No
☐ Yes
☐ Don't know

Do you perform the task of operating Plasma H2O2 Sterad* system?
☐ No
☐ Yes
☐ Don't know

How many days per week do you operate Plasma H2O2 Sterad* system?
____
(number of days per week)

How many times per day do you operate Plasma H2O2 Sterad* system?
____
(number of times per day)

Duration of task - please estimate the approximate number of minutes to operate Plasma H2O2 Sterad* system each time.
____
(number of minutes per task)

Is system closed while operating Plasma H2O2 Sterad* system?
☐ No
☐ Yes
☐ Don't know

Is local exhaust ventilation used while operating Plasma H2O2 Sterad* system?
☐ No
☐ Yes
☐ Don't know

Do you perform the task of operating Steris* system?
☐ No
☐ Yes
☐ Don't know

How many days per week do you operate Steris* system?
____
(number of days per week)

How many times per day do you operate Steris* system?
____
(number of times per day)

Duration of task - please estimate the approximate number of minutes to operate Steris* system each time.
____
(number of minutes per task)

Is system closed while operating Steris* system?
☐ No
☐ Yes
☐ Don't know

Is local exhaust ventilation used while operating Steris* system?
☐ No
☐ Yes
☐ Don't know

Do you perform the task of cleaning or replacing equipment screens or filters?
☐ No
☐ Yes
☐ Don't know

How many days per week do you clean or replace equipment screens or filters?

____
(number of days per week)

How many times per day do you clean or replace equipment screens or filters?

____
(number of times per day)

Duration of task - please estimate the approximate number of minutes to clean or replace equipment screens or filters each time.

____
(number of minutes per task)

Is system closed while cleaning or replacing equipment screens or filters?

- ☐ No
- ☐ Yes
- ☐ Don't know

Is local exhaust ventilation used while cleaning or replacing equipment screens or filters?

- ☐ No
- ☐ Yes
- ☐ Don't know

Do you rinse or flush sterilized medical instruments with alcohol?

- ☐ Yes
- ☐ No

After rinsing or flushing with alcohol do you use forced air to dry medical instruments?

- ☐ Yes
- ☐ No

What special personal protective equipment do you use when sterilizing medical equipment or instruments?

Do you wear latex gloves?

- ☐ always
- ☐ mostly
- ☐ sometimes
- ☐ never

Do you wear chemical resistant gloves?

- ☐ always
- ☐ mostly
- ☐ sometimes
- ☐ never

Do you wear a chemical resistant apron?

- ☐ always
- ☐ mostly
- ☐ sometimes
- ☐ never

Do you wear a face shield?

- ☐ always
- ☐ mostly
- ☐ sometimes
- ☐ never

Do you wear a surgical mask?

- ☐ always
- ☐ mostly
- ☐ sometimes
- ☐ never

Do you wear a N95 respirator?

- ☐ always
- ☐ mostly
- ☐ sometimes
- ☐ never

Do you wear a respirator with particulate filter cartridges?

- ☐ always
- ☐ mostly
- ☐ sometimes
- ☐ never

Do you wear a respirator with chemical cartridges?

- ☐ always
- ☐ mostly
- ☐ sometimes
- ☐ never

Have you been trained in the use of personal protective equipment?

- ☐ No
- ☐ Yes
- ☐ Don't know

Exposure to Cleaning & Disinfectant Products used on Fixed Surfaces, Equipment or Instruments (Current Job)

Do you clean or disinfect fixed surfaces, equipment or instruments such as: countertops, floors, beds, bathrooms, IV poles, monitors, carts, computers, blood pressure cuffs, and stethoscopes with cleaners or disinfectants such as bleach/chlorine, ammonia, chloramines, quats, phenolics, floor stripper, acids, detergents?

- ☐ Yes
- ☐ No

What are the names of the cleaners/disinfectants you use to clean surfaces of equipment or instruments. Please select the brand or product from the list or write in brand or product names, how many days per week you use and the type of gloves used when you use these cleaners/disinfectants.

Glass cleaning products such as Windex* - do you use this chemical or product?

- ☐ No
- ☐ Yes
- ☐ Don't know

Glass cleaning products such as Windex* - used how many days per week?

- ☐ less than 1
- ☐ 1
- ☐ 2
- ☐ 3
- ☐ 4
- ☐ 5
- ☐ 6
- ☐ 7

Glass cleaning products such as Windex* - are gloves worn with this chemical or product?

- ☐ none
- ☐ Nitrile
- ☐ Latex or Vinyl
- ☐ Don't Know

Acid - do you use this chemical or product?

- ☐ No
- ☐ Yes
- ☐ Don't know

Acid - used how many days per week?

- ☐ less than 1
- ☐ 1
- ☐ 2
- ☐ 3
- ☐ 4
- ☐ 5
- ☐ 6
- ☐ 7

Acid - are gloves worn with this chemical or product?

- ☐ none
- ☐ Nitrile
- ☐ Latex or Vinyl
- ☐ Don't Know

Alcohol such as ethanol and isopropanol - do you use this chemical or product?

- ☐ No
- ☐ Yes
- ☐ Don't know

Alcohol such as ethanol and isopropanol - used how many days per week?

- ☐ less than 1
- ☐ 1
- ☐ 2
- ☐ 3
- ☐ 4
- ☐ 5
- ☐ 6
- ☐ 7

Alcohol such as ethanol and isopropanol - are gloves worn with this chemical or product?

- ☐ none
- ☐ Nitrile
- ☐ Latex or Vinyl
- ☐ Don't Know

Ammonia - do you use this chemical or product?

- ☐ No
- ☐ Yes
- ☐ Don't know

Ammonia - used how many days per week?

- ☐ less than 1
- ☐ 1
- ☐ 2
- ☐ 3
- ☐ 4
- ☐ 5
- ☐ 6
- ☐ 7

Ammonia - are gloves worn with this chemical or product?

- ☐ none
- ☐ Nitrile
- ☐ Latex or Vinyl
- ☐ Don't Know

Bleach or chlorine such as Clorox* - do you use this chemical or product?

- ☐ No
- ☐ Yes
- ☐ Don't know

Bleach or chlorine such as Clorox* - used how many days per week?

- ☐ less than 1
- ☐ 1
- ☐ 2
- ☐ 3
- ☐ 4
- ☐ 5
- ☐ 6
- ☐ 7

Bleach or chlorine such as Clorox* - are gloves worn with this chemical or product?

- ☐ none
- ☐ Nitrile
- ☐ Latex or Vinyl
- ☐ Don't Know

Detergents - do you use this chemical or product?

- ☐ No
- ☐ Yes
- ☐ Don't know

Detergents - used how many days per week?

- ☐ less than 1
- ☐ 1
- ☐ 2
- ☐ 3
- ☐ 4
- ☐ 5
- ☐ 6
- ☐ 7

Detergents - are gloves worn with this chemical or product?

- ☐ none
- ☐ Nitrile
- ☐ Latex or Vinyl
- ☐ Don't Know

Enzymatic cleaners such as Asepti-Zyme*, 3M Rapid Multi-Enzyme* - do you use these chemicals or products?

- ☐ No
- ☐ Yes
- ☐ Don't know

Enzymatic cleaners such as Asepti-Zyme*, 3M Rapid Multi-Enzyme* - used how many days per week?

- ☐ less than 1
- ☐ 1
- ☐ 2
- ☐ 3
- ☐ 4
- ☐ 5
- ☐ 6
- ☐ 7

Enzymatic cleaners such as Asepti-Zyme*, 3M Rapid Multi-Enzyme* - are gloves worn with these chemicals or products?

- ☐ none
- ☐ Nitrile
- ☐ Latex or Vinyl
- ☐ Don't Know

Floor wax stripper - do you use this chemical or product?

- ☐ No
- ☐ Yes
- ☐ Don't know

Floor wax stripper - used how many days per week?

- ☐ less than 1
- ☐ 1
- ☐ 2
- ☐ 3
- ☐ 4
- ☐ 5
- ☐ 6
- ☐ 7

Floor wax stripper - are gloves worn with this chemical or product?

- ☐ none
- ☐ Nitrile
- ☐ Latex or Vinyl
- ☐ Don't Know

Phenolics, such as 3M Phenolic Disinfectant 18*, WexCide*, MicroBakll*, Megacide*, Novigard, Sporicidin* - do you use these chemicals or products?

- ☐ No
- ☐ Yes
- ☐ Don't know

Phenolics, such as 3M Phenolic Disinfectant 18*, WexCide*, MicroBakll*, Megacide*, Novigard*, Sporicidin* - used how many days per week?

- ☐ less than 1
- ☐ 1
- ☐ 2
- ☐ 3
- ☐ 4
- ☐ 5
- ☐ 6
- ☐ 7

Phenolics, such as 3M Phenolic Disinfectant 18*, WexCide*, MicroBakll*, Megacide*, Novigard*, Sporicidin* - are gloves worn with these chemicals or products?

- ☐ none
- ☐ Nitrile
- ☐ Latex or Vinyl
- ☐ Don't Know

Quaternary ammonium compounds "Quats", such as 3M Neutral Quat 23*, 3M HB Quat 25*, Sani-Cloth Plus*, Oasis*, Staphene*, BTC100*, BioQuat*, Sentinel* - do you use these chemicals or products?

- ☐ No
- ☐ Yes
- ☐ Don't know

Quaternary ammonium compounds "Quats", such as 3M Neutral Quat 23*, 3M HB Quat 25*, Sani-Cloth Plus*, Oasis, Staphene*, BTC100*, BioQuat*, Sentinel* - used how many days per week?

- ☐ less than 1
- ☐ 1
- ☐ 2
- ☐ 3
- ☐ 4
- ☐ 5
- ☐ 6
- ☐ 7

Quaternary ammonium compounds "Quats", such as 3M Neutral Quat 23*, 3M HB Quat 25*, Sani-Cloth Plus*, Oasis*, Staphene*, BTC100*, BioQuat*, Sentinel* - are gloves worn with these chemicals or products?

- ☐ none
- ☐ Nitrile
- ☐ Latex or Vinyl
- ☐ Don't Know

Please list any other cleaning or disinfection chemicals or products you use.

Name brand or product #1 ______________________________

Used how many days per week?
- ☐ less than 1
- ☐ 1
- ☐ 2
- ☐ 3
- ☐ 4
- ☐ 5
- ☐ 6
- ☐ 7

Are gloves worn with these chemicals or products?
- ☐ none
- ☐ Nitrile
- ☐ Latex or Vinyl
- ☐ Don't Know

Name brand or product #2 ______________________________

Used how many days per week?
- ☐ less than 1
- ☐ 1
- ☐ 2
- ☐ 3
- ☐ 4
- ☐ 5
- ☐ 6
- ☐ 7

Are gloves worn with these chemicals or products?
- ☐ none
- ☐ Nitrile
- ☐ Latex or Vinyl
- ☐ Don't Know

Name brand or product #3 ______________________________

Used how many days per week?
- ☐ less than 1
- ☐ 1
- ☐ 2
- ☐ 3
- ☐ 4
- ☐ 5
- ☐ 6
- ☐ 7

Are gloves worn with these chemicals or products?
- ☐ none
- ☐ Nitrile
- ☐ Latex or Vinyl
- ☐ Don't Know

Name brand or product #4 ______________________________

Used how many days per week?
- ☐ less than 1
- ☐ 1
- ☐ 2
- ☐ 3
- ☐ 4
- ☐ 5
- ☐ 6
- ☐ 7

Are gloves worn with these chemicals or products?
- ☐ none
- ☐ Nitrile
- ☐ Latex or Vinyl
- ☐ Don't Know

Name brand or product #5 ______________________________

Used how many days per week?
- [ ] less than 1
- [ ] 1
- [ ] 2
- [ ] 3
- [ ] 4
- [ ] 5
- [ ] 6
- [ ] 7

Are gloves worn with these chemicals or products?
- [ ] none
- [ ] Nitrile
- [ ] Latex or Vinyl
- [ ] Don't Know

Name brand or product #6 ______________________________

Used how many days per week?
- [ ] less than 1
- [ ] 1
- [ ] 2
- [ ] 3
- [ ] 4
- [ ] 5
- [ ] 6
- [ ] 7

Are gloves worn with these chemicals or products?
- [ ] none
- [ ] Nitrile
- [ ] Latex or Vinyl
- [ ] Don't Know

On a typical day when you use cleaners or disinfectants on fixed surfaces, equipment or instruments, how many times per day do you use these products?
- [ ] less than 1 time per day
- [ ] 1-3 times per day
- [ ] 4-10 times per day
- [ ] greater than 10 times per day

On a typical day when you use cleaners or disinfectants on fixed surfaces, equipment or instruments, how many hours per day do you use these products?
- [ ] less than 1 hour per day
- [ ] 1-2 hours per day
- [ ] greater than 2-4 hours per day
- [ ] greater than 4-6 hours per day
- [ ] greater than 6 hours per day

When you use cleaners or disinfectants on fixed surfaces, equipment or instruments, do you use more sprays or more wipes, or do you use both equally often? Select the ONE best answer.
- [ ] Use more sprays than wipes
- [ ] Use more wipes than sprays
- [ ] Use sprays and wipes about equally
- [ ] Not sure which I use more

What tasks do you perform and tools do you use when cleaning and disinfecting surfaces; and what surfaces do you clean and disinfect? In the questions below, please indicate how many days per week, times per week, duration of task and type of gloves used when you perform this task.

Do you wipe down beds, furniture, counters, walls, etc?
- [ ] No
- [ ] Yes
- [ ] Don't know

How many days per week?
- [ ] less than 1
- [ ] 1
- [ ] 2
- [ ] 3
- [ ] 4
- [ ] 5
- [ ] 6
- [ ] 7

How many times per day? ______________________________
(number of times per day)

Duration of task - please estimate the approximate number of minutes to wipe down beds, furniture, coutners, walls, etc.

____
(number of minutes per task)

Are gloves worn with this task?

☐ none
☐ Nitrile
☐ Latex or Vinyl
☐ Don't Know

Do you clean up spills or blood?

☐ No
☐ Yes
☐ Don't know

How many days per week?

☐ less than 1
☐ 1
☐ 2
☐ 3
☐ 4
☐ 5
☐ 6
☐ 7

How many times per day?

____
(number of times per day)

Duration of task - please estimate the approximate number of minutes to clean spills or blood.

____
(number of minutes per task)

Are gloves worn with this task?

☐ none
☐ Nitrile
☐ Latex or Vinyl
☐ Don't Know

Do you manually mix, refill, or empty cleaning or disinfecting products?

☐ No
☐ Yes
☐ Don't know

How many days per week?

☐ less than 1
☐ 1
☐ 2
☐ 3
☐ 4
☐ 5
☐ 6
☐ 7

How many times per day?

____
(number of times per day)

Duration of task - please estimate the approximate number of minutes to manually mix, refill, or empty cleaning or disinfecting products.

____
(number of minutes per task)

Are gloves worn with this task?

☐ none
☐ Nitrile
☐ Latex or Vinyl
☐ Don't Know

Do you clean bathrooms including toilet, sink, shower?

☐ No
☐ Yes
☐ Don't know

How many days per week?

☐ less than 1
☐ 1
☐ 2
☐ 3
☐ 4
☐ 5
☐ 6
☐ 7

How many times per day?

____
(number of times per day)

Duration of task - please estimate the approximate number of minutes to clean bathrooms including toilet, sink, shower.

____
(number of minutes per task)

Are gloves worn with this task?

☐ none
☐ Nitrile
☐ Latex or Vinyl
☐ Don't Know

Do you spray then wipe glass, windows, mirrors?

☐ No
☐ Yes
☐ Don't know

How many days per week?

☐ less than 1
☐ 1
☐ 2
☐ 3
☐ 4
☐ 5
☐ 6
☐ 7

How many times per day?

____
(number of times per day)

Duration of task - please estimate the approximate number of minutes to spray then wipe glass, windows, mirrors.

____
(number of minutes per task)

Are gloves worn with this task?

☐ none
☐ Nitrile
☐ Latex or Vinyl
☐ Don't Know

Do you polish wood furniture?

☐ No
☐ Yes
☐ Don't know

How many days per week?

☐ less than 1
☐ 1
☐ 2
☐ 3
☐ 4
☐ 5
☐ 6
☐ 7

How many times per day?

____
(number of times per day)

Duration of task - please estimate the approximate number of minutes to polish wood furniture.

____
(number of minutes per task)

Are gloves worn with this task?	☐ none<br>☐ Nitrile<br>☐ Latex or Vinyl<br>☐ Don't Know
Do you polish stainless steel surfaces?	☐ No<br>☐ Yes<br>☐ Don't know
How many days per week?	☐ less than 1<br>☐ 1<br>☐ 2<br>☐ 3<br>☐ 4<br>☐ 5<br>☐ 6<br>☐ 7
How many times per day?	______________________<br>(number of times per day)
Duration of task - please estimate the approximate number of minutes to polish stainless steel surfaces.	______________________<br>(number of minutes per task)
Are gloves worn with this task?	☐ none<br>☐ Nitrile<br>☐ Latex or Vinyl<br>☐ Don't Know
Do you spray deodorant/disinfectant?	☐ No<br>☐ Yes<br>☐ Don't know
How many days per week?	☐ less than 1<br>☐ 1<br>☐ 2<br>☐ 3<br>☐ 4<br>☐ 5<br>☐ 6<br>☐ 7
How many times per day?	______________________<br>(number of times per day)
Duration of task - please estimate the approximate number of minutes to spray deodorant/disinfectant.	______________________<br>(number of minutes per task)
Are gloves worn with this task?	☐ none<br>☐ Nitrile<br>☐ Latex or Vinyl<br>☐ Don't Know
Do you mop floors?	☐ No<br>☐ Yes<br>☐ Don't know
How many days per week?	☐ less than 1<br>☐ 1<br>☐ 2<br>☐ 3<br>☐ 4<br>☐ 5<br>☐ 6<br>☐ 7
How many times per day?	______________________<br>(number of times per day)

Duration of task - please estimate the approximate number of minutes to mop floors.

____
(number of minutes per task)

Are gloves worn with this task?

- ☐ none
- ☐ Nitrile
- ☐ Latex or Vinyl
- ☐ Don't Know

Do you remove soiled linen, trash, or containers contaminated with blood, vomit, urine, feces, or chemicals?

- ☐ No
- ☐ Yes
- ☐ Don't know

How many days per week?

- ☐ less than 1
- ☐ 1
- ☐ 2
- ☐ 3
- ☐ 4
- ☐ 5
- ☐ 6
- ☐ 7

How many times per day?

____
(number of times per day)

Duration of task - please estimate the approximate number of minutes to remove soiled linen, trash, or containers contaminated with blood, vomit, urine, feces, or chemicals.

____
(number of minutes per task)

Are gloves worn with this task?

- ☐ none
- ☐ Nitrile
- ☐ Latex or Vinyl
- ☐ Don't Know

Do you clean instruments such as scissors, stethoscopes, and thermometers?

- ☐ No
- ☐ Yes
- ☐ Don't know

How many days per week?

- ☐ less than 1
- ☐ 1
- ☐ 2
- ☐ 3
- ☐ 4
- ☐ 5
- ☐ 6
- ☐ 7

How many times per day?

____
(number of times per day)

Duration of task - please estimate the approximate number of minutes to clean instruments such as scissors, stethoscopes, and thermometers.

____
(number of minutes per task)

Are gloves worn with this task?

- ☐ none
- ☐ Nitrile
- ☐ Latex or Vinyl
- ☐ Don't Know

Do you clean equipment such as IV poles, carts, monitors and computers?

- ☐ No
- ☐ Yes
- ☐ Don't know

How many days per week?

- ☐ less than 1
- ☐ 1
- ☐ 2
- ☐ 3
- ☐ 4
- ☐ 5
- ☐ 6
- ☐ 7

How many times per day?

____
(number of times per day)

Duration of task - please estimate the approximate number of minutes to clean equipment such as IV poles, carts, monitors and computers.

____
(number of minutes per task)

Are gloves worn with this task?

- ☐ none
- ☐ Nitrile
- ☐ Latex or Vinyl
- ☐ Don't Know

Do you do terminal cleaning of patient rooms?

- ☐ No
- ☐ Yes
- ☐ Don't know

How many days per week?

- ☐ less than 1
- ☐ 1
- ☐ 2
- ☐ 3
- ☐ 4
- ☐ 5
- ☐ 6
- ☐ 7

How many times per day?

____
(number of times per day)

Duration of task - please estimate the approximate number of minutes to clean rooms of terminally ill patients.

____
(number of minutes per task)

Are gloves worn with this task?

- ☐ none
- ☐ Nitrile
- ☐ Latex or Vinyl
- ☐ Don't Know

Do you clean or disinfect rooms of patients with MRSA, VRE or other drug resistant bacteria in patient rooms?

- ☐ No
- ☐ Yes
- ☐ Don't know

How many days per week?

- ☐ less than 1
- ☐ 1
- ☐ 2
- ☐ 3
- ☐ 4
- ☐ 5
- ☐ 6
- ☐ 7

How many times per day?

____
(number of times per day)

Duration of task - please estimate the approximate number of minutes to clean or disinfect for MRSA, VRE or other drug resistant bacteria in patient rooms.

____
(number of minutes per task)

Are gloves worn with this task?

☐ none
☐ Nitrile
☐ Latex or Vinyl
☐ Don't Know

Do you do end of day cleaning of operating rooms, dialysis units or other patient care areas?

☐ No
☐ Yes
☐ Don't know

How many days per week?

☐ less than 1
☐ 1
☐ 2
☐ 3
☐ 4
☐ 5
☐ 6
☐ 7

How many times per day?

____
(number of times per day)

Duration of task - please estimate the approximate number of minutes to clean operating rooms, diaysis units or other patient care areas at the end of shift.

____
(number of minutes per task)

Are gloves worn with this task?

☐ none
☐ Nitrile
☐ Latex or Vinyl
☐ Don't Know

Do you strip, wax and buff floors using strippers and waxes?

☐ Yes
☐ No

What tasks do you perform and tools do you use to strip, wax and buff floors? Please indicate how many days per week, times per week, duration of task and the type of gloves used when you perform this task.

Do you strip floors?

☐ No
☐ Yes
☐ Don't know

How many days per week?

☐ less than 1
☐ 1
☐ 2
☐ 3
☐ 4
☐ 5
☐ 6
☐ 7

How many times per day?

____
(number of times per day)

Duration of task - please estimate the approximate number of minutes to strip floors.

____
(number of minutes per task)

Are gloves worn with this task?

☐ none
☐ Nitrile
☐ Latex or Vinyl
☐ Don't Know

Do you scrape floors?

☐ No
☐ Yes
☐ Don't know

How many days per week?

☐ less than 1
☐ 1
☐ 2
☐ 3
☐ 4
☐ 5
☐ 6
☐ 7

How many times per day?

____
(number of times per day)

Duration of task - please estimate the approximate number of minutes to scrape floors.

____
(number of minutes per task)

Are gloves worn with this task?

☐ none
☐ Nitrile
☐ Latex or Vinyl
☐ Don't Know

Do you buff floors?

☐ No
☐ Yes
☐ Don't know

How many days per week?

☐ less than 1
☐ 1
☐ 2
☐ 3
☐ 4
☐ 5
☐ 6
☐ 7

How many times per day?

____
(number of times per day)

Duration of task - please estimate the approximate number of minutes to buff floors.

____
(number of minutes per task)

Are gloves worn with this task?

☐ none
☐ Nitrile
☐ Latex or Vinyl
☐ Don't Know

Do you wax floors?

☐ No
☐ Yes
☐ Don't know

How many days per week?

☐ less than 1
☐ 1
☐ 2
☐ 3
☐ 4
☐ 5
☐ 6
☐ 7

How many times per day?

____
(number of times per day)

Duration of task - please estimate the approximate number of minutes to wax floors.

____
(number of minutes per task)

Are gloves worn with this task?

☐ none
☐ Nitrile
☐ Latex or Vinyl
☐ Don't Know

What special personal protective equipment do you use when stripping or waxing floors or cleaning surfaces?

Do you wear a chemical resistant apron?
☐ always
☐ mostly
☐ sometimes
☐ never

Do you wear a face shield?
☐ always
☐ mostly
☐ sometimes
☐ never

Do you wear a surgical mask?
☐ always
☐ mostly
☐ sometimes
☐ never

Do you wear a N95 respirator?
☐ always
☐ mostly
☐ sometimes
☐ never

Do you wear a respirator with particulate filter cartridges?
☐ always
☐ mostly
☐ sometimes
☐ never

Do you wear a respirator with chemical cartridges?
☐ always
☐ mostly
☐ sometimes
☐ never

Have you been trained in the use of personal protective equipment?
☐ no
☐ yes
☐ don't know

Are there any other exposure control measures when you clean surfaces?

Is the mixing system enclosed?
☐ No
☐ Yes
☐ N/A

Does the room where you clean surfaces have vents in the ceiling?
☐ No
☐ Yes
☐ N/A

Do you turn on the fan when you clean bathrooms?
☐ No
☐ Yes
☐ N/A

Exposure to Chemicals used in Medical/Clinical or Dental Laboratory, Radiology, or Pharmacy in Patient/Chemical Preparation Area (Current Job)

Do you use chemicals, medicines, solvents, developing solution or metals (such as xylenes, methylmethacrylates, mercury, beryllium) at work in the laboratory or in patient/chemical preparation areas?
☐ Yes
☐ No

Do you work in the Clinical, Histology, Microbiology, or Pathology laboratories?
☐ Yes
☐ No

Do you use formalin to fix grossed tissue and autopsy specimens.
☐ No
☐ Yes
☐ Don't know

How many days per week?

☐ less than 1
☐ 1
☐ 2
☐ 3
☐ 4
☐ 5
☐ 6
☐ 7

How many times per day?

____
(number of times per day)

Duration of task - please estimate the approximate number of minutes to fix grossed tissue and autopsy specimens.

____
(number of minutes per task)

Do you perform these tasks in a fume hood?

☐ No
☐ Yes
☐ Don't know

Do you perform these tasks using bench-top local exhaust ventilation?

☐ No
☐ Yes
☐ Don't Know

Do you use stains and dyes such as haematoxylin and eosin stain?

☐ No
☐ Yes
☐ Don't know

How many days per week?

☐ less than 1
☐ 1
☐ 2
☐ 3
☐ 4
☐ 5
☐ 6
☐ 7

How many times per day?

____
(number of times per day)

Duration of task - please estimate the approximate number of minutes you use stains and dyes such as haematoxylin and eosin stain.

____
(number of minutes per task)

Do you perform these tasks in a fume hood?

☐ No
☐ Yes
☐ Don't know

Do you perform these tasks using bench-top local exhaust ventilation?

☐ No
☐ Yes
☐ Don't Know

Do you use solvents such as xylene and toluene to fix tissue specimens and rinse stains?

☐ No
☐ Yes
☐ Don't know

How many days per week?

☐ less than 1
☐ 1
☐ 2
☐ 3
☐ 4
☐ 5
☐ 6
☐ 7

How many times per day?

____
(number of times per day)

Duration of task - please estimate the approximate number of minutes you use solvents such as xylene and toluene to fix tissue specimens and rinse stains.

__________________________ (number of minutes per task)

Do you perform these tasks in a fume hood?

☐ No
☐ Yes
☐ Don't know

Do you perform these tasks using bench-top local exhaust ventilation?

☐ No
☐ Yes
☐ Don't Know

Do you work in the Dental Laboratories?

☐ Yes
☐ No

Do you mix plaster, acrylic pastes (methyl methacrylates) or glues for dentures?

☐ No
☐ Yes
☐ Don't know

How many days per week?

☐ less than 1
☐ 1
☐ 2
☐ 3
☐ 4
☐ 5
☐ 6
☐ 7

How many times per day?

__________________________ (number of times per day)

Duration of task - please estimate the approximate number of minutes you mix plaster, acrylic pastes (methyl methacrylates) or glues for dentures.

__________________________ (number of minutes per task)

Do you perform these tasks in a fume hood?

☐ No
☐ Yes
☐ Don't know

Do you perform these tasks using bench-top local exhaust ventilation?

☐ No
☐ Yes
☐ Don't Know

Do you prepare, mix, pour resins, solutions, chemicals and solvents?

☐ No
☐ Yes
☐ Don't know

How many days per week?

☐ less than 1
☐ 1
☐ 2
☐ 3
☐ 4
☐ 5
☐ 6
☐ 7

How many times per day?

__________________________ (number of times per day)

Duration of task - please estimate the approximate number of minutes you prepare, mix, pour resins, solutions, chemicals and solvents.

__________________________ (number of minutes per task)

Do you perform these tasks in a fume hood?

☐ No
☐ Yes
☐ Don't know

Do you perform these tasks using bench-top local exhaust ventilation?
- ☐ No
- ☐ Yes
- ☐ Don't Know

Do you cast metal dental frameworks using alloys such as BE, Cr, CO and NI?
- ☐ No
- ☐ Yes
- ☐ Don't know

How many days per week?
- ☐ less than 1
- ☐ 1
- ☐ 2
- ☐ 3
- ☐ 4
- ☐ 5
- ☐ 6
- ☐ 7

How many times per day?

____
(number of times per day)

Duration of task - please estimate the approximate number of minutes you cast metal dental frameworks using alloys such as BE, Cr, CO and NI.

____
(number of minutes per task)

Do you perform these tasks in a fume hood?
- ☐ No
- ☐ Yes
- ☐ Don't know

Do you perform these tasks using bench-top local exhaust ventilation?
- ☐ No
- ☐ Yes
- ☐ Don't Know

Do you prepare metal surfaces by cutting, grinding and sand blasting?
- ☐ No
- ☐ Yes
- ☐ Don't know

How many days per week?
- ☐ less than 1
- ☐ 1
- ☐ 2
- ☐ 3
- ☐ 4
- ☐ 5
- ☐ 6
- ☐ 7

How many times per day?

____
(number of times per day)

Duration of task - please estimate the approximate number of minutes you prepare metal surfaces by cutting, grinding and sand blasting.

____
(number of minutes per task)

Do you perform these tasks in a fume hood?
- ☐ No
- ☐ Yes
- ☐ Don't know

Do you perform these tasks using bench-top local exhaust ventilation?
- ☐ No
- ☐ Yes
- ☐ Don't Know

Do you apply porcelain layer and bake and grind?
- ☐ No
- ☐ Yes
- ☐ Don't know

How many days per week?

- [ ] less than 1
- [ ] 1
- [ ] 2
- [ ] 3
- [ ] 4
- [ ] 5
- [ ] 6
- [ ] 7

How many times per day?

____
(number of times per day)

Duration of task - please estimate the approximate number of minutes you apply porcelain layer and bake and grind.

____
(number of minutes per task)

Do you perform these tasks in a fume hood?

- [ ] No
- [ ] Yes
- [ ] Don't know

Do you perform these tasks using bench-top local exhaust ventilation?

- [ ] No
- [ ] Yes
- [ ] Don't Know

Do you grind/polish dentures?

- [ ] No
- [ ] Yes
- [ ] Don't know

How many days per week?

- [ ] less than 1
- [ ] 1
- [ ] 2
- [ ] 3
- [ ] 4
- [ ] 5
- [ ] 6
- [ ] 7

How many times per day?

____
(number of times per day)

Duration of task - please estimate the approximate number of minutes you grind/polish dentures.

____
(number of minutes per task)

Do you perform these tasks in a fume hood?

- [ ] No
- [ ] Yes
- [ ] Don't know

Do you perform these tasks using bench-top local exhaust ventilation?

- [ ] No
- [ ] Yes
- [ ] Don't Know

Do you work in Pharmacy?

- [ ] Yes
- [ ] No

Do you mix and compound antineoplastic and other cytotoxic drugs?

- [ ] No
- [ ] Yes
- [ ] Don't know

How many days per week?

- [ ] less than 1
- [ ] 1
- [ ] 2
- [ ] 3
- [ ] 4
- [ ] 5
- [ ] 6
- [ ] 7

How many times per day?

____
(number of times per day)

Duration of task - please estimate the approximate number of minutes you mix and compound antineoplastic and other cytotoxic drugs.

____
(number of minutes per day)

Do you perform these tasks in a fume hood?

- ☐ No
- ☐ Yes
- ☐ Don't know

Do you perform these tasks using bench-top local exhaust ventilation?

- ☐ No
- ☐ Yes
- ☐ Don't Know

Do you work in the Radiology Department?

- ☐ Yes
- ☐ No

Do you handle film developing solutions?

- ☐ No
- ☐ Yes
- ☐ Don't know

How many days per week?

- ☐ less than 1
- ☐ 1
- ☐ 2
- ☐ 3
- ☐ 4
- ☐ 5
- ☐ 6
- ☐ 7

How many times per day?

____
(number of times per day)

Duration of task - please estimate the approximate number of minutes you handle film developing solutions.

____
(number of minutes per task)

Do you perform these tasks in a fume hood?

- ☐ No
- ☐ Yes
- ☐ Don't know

Do you perform these tasks using bench-top local exhaust ventilation?

- ☐ No
- ☐ Yes
- ☐ Don't Know

What special personal protective equipment do you use when you handle or use materials in the Radiology Department or Laboratories mentioned above (Histology, Microbiology, Pathology, Dental, Pharmacy)?

Do you wear latex gloves?

- ☐ always
- ☐ mostly
- ☐ sometimes
- ☐ never

Do you wear chemical resistant gloves?

- ☐ always
- ☐ mostly
- ☐ sometimes
- ☐ never

Do you wear a chemical resistant apron?

- ☐ always
- ☐ mostly
- ☐ sometimes
- ☐ never

Do you wear a face shield?

- ☐ always
- ☐ mostly
- ☐ sometimes
- ☐ never

Do you wear a surgical mask?

- ☐ always
- ☐ mostly
- ☐ sometimes
- ☐ never

Do you wear a N95 respirator?

- ☐ always
- ☐ mostly
- ☐ sometimes
- ☐ never

Do you wear a respirator with particulate filter cartridges?

- ☐ always
- ☐ mostly
- ☐ sometimes
- ☐ never

Do you wear a respirator with chemical cartridges?

- ☐ always
- ☐ mostly
- ☐ sometimes
- ☐ never

Have you been trained in the use of personal protective equipment?

- ☐ No
- ☐ Yes
- ☐ Don't Know

Exposure to Chemicals Used on Patients (Current Job)

Do you use chemicals, adhesives, antiseptics, alcohols or solvents such as iodine, hydrogen peroxide, super glue, bone cement, alcohols, alchohol preps, mineral spirits, toluene, solutions to remove adhesives at work on patients?

- ☐ Yes
- ☐ No

What tasks do you perform when you apply or use chemicals, antiseptics, adhesives, alcohols, or solvents on patients during procedures? Please indicate how many days per week, times per day, duration of task and type of gloves used.

Do you use antiseptics such as alcohols, iodine, acetic acid to disinfect skin areas on patients prior to procedure using wipes, gauze or swabs?

- ☐ No
- ☐ Yes
- ☐ Don't know

How many days per week?

- ☐ less than 1
- ☐ 1
- ☐ 2
- ☐ 3
- ☐ 4
- ☐ 5
- ☐ 6
- ☐ 7

How many times per day?

____
(number of times per day)

Duration of task - please estimate the approximate number of minutes to use antiseptics such as alcohols, iodine, acetic acid to disinfect skin areas on patients prior to procedure using wipes, gauze or swabs.

____
(number of minutes per task)

Are gloves worn with this task?

- ☐ none
- ☐ Nitrile
- ☐ Latex or Vinyl
- ☐ Don't Know

Do you clean and disinfect wounds using antiseptics such as silver compounds, chlorhexidine, povidone iodine or cadexomer iodine?

- ☐ No
- ☐ Yes
- ☐ Don't know

How many days per week?

☐ less than 1
☐ 1
☐ 2
☐ 3
☐ 4
☐ 5
☐ 6
☐ 7

How many times per day?

____
(number of times per day)

Duration of task - please estimate the approximate number of minutes to clean and disinfect wounds using antiseptics such as silver compounds, chlorhexidine, povidone iodine or cadexomer iodine.

____
(number of minutes per task)

Are gloves worn with this task?

☐ none
☐ Nitrile
☐ Latex or Vinyl
☐ Don't Know

Do you apply wound dressing such as polyurethane based hydrogel, hydrocolloid, or hydrocellular foam?

☐ No
☐ Yes
☐ Don't know

How many days per week?

☐ less than 1
☐ 1
☐ 2
☐ 3
☐ 4
☐ 5
☐ 6
☐ 7

How many times per day?

____
(number of times per day)

Duration of task - please estimate the approximate number of minutes to apply wound dressing such as polyurethane based hydrogel, hydrocolloid, or hydrocellular foam.

____
(number of minutes per task)

Are gloves worn with this task?

☐ none
☐ Nitrile
☐ Latex or Vinyl
☐ Don't Know

Do you use adhesive such as super glue, acrylates, bone cements, benzoin tincture such as 3M*, Steri-strip* for surgery, skin closure, bone repair, ostomy bags and other applications.

☐ No
☐ Yes
☐ Don't know

How many days per week?

☐ less than 1
☐ 1
☐ 2
☐ 3
☐ 4
☐ 5
☐ 6
☐ 7

How many times per day?

____
(number of times per day)

Duration of task - please estimate the approximate number of minutes to use adhesive such as super glue, acrylates, bone cements, benzoin tincture such as 3M*, Steri-strip* for surgery, skin closure, bone repair, ostomy bags and other applications.

____
(number of minutes per task)

Are gloves worn with this task?

☐ none
☐ Nitrile
☐ Latex or Vinyl
☐ Don't Know

Do you use adhesive removing solvents such as alcohols, acetone with wipes, gauze or swabs?

☐ No
☐ Yes
☐ Don't know

How many days per week?

☐ less than 1
☐ 1
☐ 2
☐ 3
☐ 4
☐ 5
☐ 6
☐ 7

How many times per day?

____
(number of times per day)

Duration of task - please estimate the approximate number of minutes to use adhesive removing solvents such as alcohols, acetone with wipes, gauze or swabs.

____
(number of minutes per task)

Are gloves worn with this task?

☐ none
☐ Nitrile
☐ Latex or Vinyl
☐ Don't Know

Do you apply casting material containing isocynates?

☐ No
☐ Yes
☐ Don't know

How many days per week?

☐ less than 1
☐ 1
☐ 2
☐ 3
☐ 4
☐ 5
☐ 6
☐ 7

How many times per day?

____
(number of times per day)

Duration of task - please estimate the approximate number of minutes to apply casting material containing isocynates.

____
(number of minutes per task)

Are gloves worn with this task?

☐ none
☐ Nitrile
☐ Latex or Vinyl
☐ Don't Know

Are you exposed to surgical smoke during laser surgery or electrosurgery?

☐ No
☐ Yes
☐ Don't know

How many days per week?

- [ ] less than 1
- [ ] 1
- [ ] 2
- [ ] 3
- [ ] 4
- [ ] 5
- [ ] 6
- [ ] 7

How many times per day?

____
(number of times per day)

Duration of task - please estimate the approximate number of minutes you are exposed to surgical smoke during laser surgery or electrosurgery.

____
(number of minutes per task)

Are gloves worn with this task?

- [ ] none
- [ ] Nitrile
- [ ] Latex or Vinyl
- [ ] Don't Know

Are you exposed to anesthetic gases during surgical procedures?

- [ ] No
- [ ] Yes
- [ ] Don't know

How many days per week?

- [ ] less than 1
- [ ] 1
- [ ] 2
- [ ] 3
- [ ] 4
- [ ] 5
- [ ] 6
- [ ] 7

How many times per day?

____
(number of times per day)

Duration of task - please estimate the approximate number of minutes you are exposed to anesthetic gases during surgical procedures

____
(number of minutes per task)

Are gloves worn with this task?

- [ ] none
- [ ] Nitrile
- [ ] Latex or Vinyl
- [ ] Don't Know

What special personal protective equipment do you use when you use chemicals/adhesives/alcohols/solvents with patients?

Do you wear a chemical resistant apron?

- [ ] always
- [ ] mostly
- [ ] sometimes
- [ ] never

Do you wear a face shield?

- [ ] always
- [ ] mostly
- [ ] sometimes
- [ ] never

Do you wear a surgical mask?

- [ ] always
- [ ] mostly
- [ ] sometimes
- [ ] never

Do you wear a N95 respirator?

- [ ] always
- [ ] mostly
- [ ] sometimes
- [ ] never

Do you wear a respirator with particulate filter cartridges?

- ☐ always
- ☐ mostly
- ☐ sometimes
- ☐ never

Do you wear a respirator with chemical cartridges?

- ☐ always
- ☐ mostly
- ☐ sometimes
- ☐ never

Have you been trained in the use of personal protective equipment?

- ☐ No
- ☐ Yes
- ☐ Don't Know

Exposure to Aerosolized Medicines Used with Patients (Current Job)

Do you administer aerosolized medications that might include antibiotics, such as Tobramycin, Amikacin, Colistin, pentamidine, ribavirin, bronchodilators, anesthetics, and antitrypsin, etc?

- ☐ Yes
- ☐ No

What tasks and tools do you use to administer aerosolized medications? Please indicate how many days per week, times per week, duration of task and the type of gloves used when you administer aerosolized medications.

Do you administer aerosolized medications with a small volume nebulizer (SVN)?

- ☐ No
- ☐ Yes
- ☐ Don't know

How many days per week?

- ☐ less than 1
- ☐ 1
- ☐ 2
- ☐ 3
- ☐ 4
- ☐ 5
- ☐ 6
- ☐ 7

How many times per day?

____
(number of times per day)

Duration of task - please estimate the approximate number of minutes you administer aerosolized medications with a small volume nebulizer (SVN).

____
(number of minutes per task)

Are gloves worn with this task?

- ☐ none
- ☐ Nitrile
- ☐ Latex or Vinyl
- ☐ Don't Know

Do you use continuous aerosol delivery system for bronchodilators and other medicines?

- ☐ No
- ☐ Yes
- ☐ Don't know

How many days per week?

- ☐ less than 1
- ☐ 1
- ☐ 2
- ☐ 3
- ☐ 4
- ☐ 5
- ☐ 6
- ☐ 7

How many times per day?

____
(number of times per day)

Duration of task - please estimate the approximate number of minutes you use continuous aerosol delivery system for bronchodilators and other medicines.

____
(number of minutes per task)

Are gloves worn with this task?
- ☐ none
- ☐ Nitrile
- ☐ Latex or Vinyl
- ☐ Don't Know

Do you administer aerosolized medications with a metered-dose inhaler (MDI)?
- ☐ No
- ☐ Yes
- ☐ Don't know

How many days per week?
- ☐ less than 1
- ☐ 1
- ☐ 2
- ☐ 3
- ☐ 4
- ☐ 5
- ☐ 6
- ☐ 7

How many times per day?

____
(number of times per day)

Duration of task - please estimate the approximate number of minutes you administer aerosolized medications with a metered-dose inhaler (MDI).

____
(number of minutes per task)

Are gloves worn with this task?
- ☐ none
- ☐ Nitrile
- ☐ Latex or Vinyl
- ☐ Don't Know

Do you administer aerosolized medications with a dry powder inhaler (DPI)?
- ☐ No
- ☐ Yes
- ☐ Don't know

How many days per week?
- ☐ less than 1
- ☐ 1
- ☐ 2
- ☐ 3
- ☐ 4
- ☐ 5
- ☐ 6
- ☐ 7

How many times per day?

____
(number of times per day)

Duration of task - please estimate the approximate number of minutes you administer aerosolized medications with a dry powder inhaler (DPI).

____
(number of minutes per task)

Are gloves worn with this task?
- ☐ none
- ☐ Nitrile
- ☐ Latex or Vinyl
- ☐ Don't Know

How do you apply or use these medicines with patients?

Do you handle medicines during preparation or cleanup?
- ☐ Yes
- ☐ No

Does the nebulizer have filters or scavengers to capture excess medicine?
- ☐ Yes
- ☐ No

Does the nebulizer have a ventilation system to capture excess medicine?
- ☐ Yes
- ☐ No

Is the nebulizing machine located in a negative pressure room?
- ☐ Yes
- ☐ No

What special personal protective equipment do you use when you administer aerosolized medicines?

Do you wear a chemical resistant apron?
☐ always
☐ mostly
☐ sometimes
☐ never

Do you wear a face shield?
☐ always
☐ mostly
☐ sometimes
☐ never

Do you wear a surgical mask?
☐ always
☐ mostly
☐ sometimes
☐ never

Do you wear a N95 respirator?
☐ always
☐ mostly
☐ sometimes
☐ never

Do you wear a respirator with particulate filter cartridges?
☐ always
☐ mostly
☐ sometimes
☐ never

Do you wear a respirator with chemical cartridges?
☐ always
☐ mostly
☐ sometimes
☐ never

Have you been trained in the use of personal protective equipment?
☐ No
☐ Yes
☐ Don't Know

Exposure to Chemicals used by Dental Assistants and Hygienists (Current Job)

Have you ever worked as a dental assistant or a hygienist?
☐ Yes
☐ No

What tasks do you do as a dental assistant or hygienist? Please indicate how many days per week, times per week, duration of task and the type of gloves used when you do these tasks.

Do you adjust, polish or repair dentures or use compunds such as methyl methacrylates, other acrylates and epoxys?
☐ No
☐ Yes
☐ Don't know

How many days per week?
☐ less than 1
☐ 1
☐ 2
☐ 3
☐ 4
☐ 5
☐ 6
☐ 7

How many times per day?
____
(number of times per day)

Duration of task - please estimate the approximate number of minutes you adjust, polish or repair dentures or use compunds such as methyl methacrylates, other acrylates and epoxys.
____
(number of minutes per task)

Are gloves worn with this task?

☐ none
☐ Nitrile
☐ Latex or Vinyl
☐ Don't Know

Do you prepare emalgams such as Vertex SoftA*, Villacryl SoftA*, Molloplast BA* and MollosilA*?

☐ No
☐ Yes
☐ Don't know

How many days per week?

☐ less than 1
☐ 1
☐ 2
☐ 3
☐ 4
☐ 5
☐ 6
☐ 7

How many times per day?

____
(number of times per day)

Duration of task - please estimate the approximate number of minutes you prepare emalgams such as Vertex SoftA*, Villacryl SoftA, Molloplast BA* and MollosilA.

____
(number of minutes per task)

Are gloves worn with this task?

☐ none
☐ Nitrile
☐ Latex or Vinyl
☐ Don't Know

Do you develop x-rays using film developing solutions?

☐ No
☐ Yes
☐ Don't know

How many days per week?

☐ less than 1
☐ 1
☐ 2
☐ 3
☐ 4
☐ 5
☐ 6
☐ 7

How many times per day?

____
(number of times per day)

Duration of task - please estimate the approximate number of minutes you develop x-rays using film developing solutions.

____
(number of minutes per task)

Are gloves worn with this task?

☐ none
☐ Nitrile
☐ Latex or Vinyl
☐ Don't Know

Do you assist or demonstrate denture placement using adhesives?

☐ No
☐ Yes
☐ Don't know

How many days per week?

☐ less than 1
☐ 1
☐ 2
☐ 3
☐ 4
☐ 5
☐ 6
☐ 7

How many times per day? ______________________ (number of times per day)

Duration of task - please estimate the approximate number of minutes you assist or demonstrate denture placement using adhesives. ______________________ (number of minutes per task)

Are gloves worn with this task?
- ☐ none
- ☐ Nitrile
- ☐ Latex or Vinyl
- ☐ Don't Know

What special personal protective equipment do you use when you handle or use these dental materials?

Do you wear a chemical resistant apron?
- ☐ always
- ☐ mostly
- ☐ sometimes
- ☐ never

Do you wear a face shield?
- ☐ always
- ☐ mostly
- ☐ sometimes
- ☐ never

Do you wear a surgical mask?
- ☐ always
- ☐ mostly
- ☐ sometimes
- ☐ never

Do you wear a N95 respirator?
- ☐ always
- ☐ mostly
- ☐ sometimes
- ☐ never

Do you wear a respirator with particulate filter cartridges?
- ☐ always
- ☐ mostly
- ☐ sometimes
- ☐ never

Do you wear a respirator with chemical cartridges?
- ☐ always
- ☐ mostly
- ☐ sometimes
- ☐ never

Have you been trained in the use of personal protective equipment?
- ☐ No
- ☐ Yes
- ☐ Don't Know

Exposure to Accidental Chemical Spill or Gas Release at Work

Have you been exposed directly or indirectly (nearby observer) to any accidental chemical spills or gas release in your workplace?
- ☐ Yes
- ☐ No

How many times in the past 10 years have you been exposed to any accidental chemical spills or gas release in your workplace? ______________________

In what month/year did the worst accidental chemical spill or gas release that you were exposed to occur? ______________________ (month-2 digits/year-4 digits (e.g. 06/2006))

Did you have to receive medical attention because of the most recent accidental exposure?
- ☐ Yes
- ☐ No

(name of chemical or gas)

What were you exposed to? ______________________

SECTION V: EXPOSURES AT HOME

Exposure to Mold, Moisture at Home

Have you observed any of the following in your house or apartment?

Water leakage or water damage indoors on walls, floors, carpets or ceilings?

In the past 2 years? ☐ Yes ☐ No

In the past 6 months? ☐ Yes ☐ No

In the past 4 weeks? ☐ Yes ☐ No

Roof leaks or plumbing leaks in kitchen or bathroom?

In the past 2 years? ☐ Yes ☐ No

In the past 6 months? ☐ Yes ☐ No

In the past 4 weeks? ☐ Yes ☐ No

Visible mold growth (not on food) indoors on walls, floors, or ceilings or in the basement?

In the past 2 years? ☐ Yes ☐ No

In the past 6 months? ☐ Yes ☐ No

In the past 4 weeks? ☐ Yes ☐ No

Visible mold growth in your bathroom on tiles, tub surround, ceiling, walls, shower curtain?

In the past 2 years? ☐ Yes ☐ No

In the past 6 months? ☐ Yes ☐ No

In the past 4 weeks? ☐ Yes ☐ No

Odor of mold or mildew or musty smell (not from food) in the house or in the basement?

In the past 2 years? ☐ Yes ☐ No

In the past 6 months? ☐ Yes ☐ No

In the past 4 weeks? ☐ Yes ☐ No

Construction at Home

Have there been any renovations or construction in your house or apartment?

In the past 2 years?
- [ ] Yes
- [ ] No

In the past 6 months?
- [ ] Yes
- [ ] No

In the past 4 weeks?
- [ ] Yes
- [ ] No

Was the renovation related to water damage or mold?
- [ ] Yes
- [ ] No

Painting walls and fixtures?

In the past 2 years?
- [ ] Yes
- [ ] No

In the past 6 months?
- [ ] Yes
- [ ] No

In the past 4 weeks?
- [ ] Yes
- [ ] No

Ripping out and replacing walls, woodwork, and partitions?

In the past 2 years?
- [ ] Yes
- [ ] No

In the past 6 months?
- [ ] Yes
- [ ] No

In the past 4 weeks?
- [ ] Yes
- [ ] No

Ripping out and replacing carpets and fixed furniture?

In the past 2 years?
- [ ] Yes
- [ ] No

In the past 6 months?
- [ ] Yes
- [ ] No

In the past 4 weeks?
- [ ] Yes
- [ ] No

Exposure to Chemicals and Cleaning Products at Home

How often do you personally clean your own home?
- [ ] Never
- [ ] less than 1
- [ ] 1-2
- [ ] 2-4
- [ ] greater than 4

(Number of days per week)

How many days a week do you use the following cleaning products in your own home?

Ammonia
- [ ] Never
- [ ] less than 1
- [ ] 1-2
- [ ] 2-4
- [ ] greater than 4

(Number of days per week)

Bleach

☐ Never
☐ less than 1
☐ 1-2
☐ 2-4
☐ greater than 4
(Number of days per week)

Spray Cleaning Products

☐ Never
☐ less than 1
☐ 1-2
☐ 2-4
☐ greater than 4
(Number of days per week)

Air Freshening Sprays (like Febreze, Glade)

☐ Never
☐ less than 1
☐ 1-2
☐ 2-4
☐ greater than 4
(Number of days per week)

Additional Product 1 Name ______________________________

Additional Product 1 Number of Days

☐ less than 1
☐ 1-2
☐ 2-4
☐ greater than 4
(Number of days per week)

Additional Product 2 Name ______________________________

Additional Product 2 Number of Days

☐ less than 1
☐ 1-2
☐ 2-4
☐ greater than 4
(Number of days per week)

Additional Product 3 Name ______________________________

Additional Product 3 Number of Days

☐ less than 1
☐ 1-2
☐ 2-4
☐ greater than 4
(Number of days per week)

Additional Product 4 Name ______________________________

Additional Product 4 Number of Days

☐ less than 1
☐ 1-2
☐ 2-4
☐ greater than 4
(Number of days per week)

SECTION VI: JOB RELOCATION INFORMATION Work History Information (last 10 years)

Have you ever had to change your job, job duties, or job location because the job affected your breathing?

☐ Yes
☐ No

When was this change in job, job duties, or job location? ______________________________
(month-2 digits/year-4 digits (e.g. 06/2006))

What was your job before the change? ______________________________

After the change, what was different?

Job ______________________

Job duties ______________________

Location ______________________

Use of or exposure to:
☐ Medications
☐ Cleaning Products
☐ Other chemicals
☐ Other factors
(check all that apply)

With this change, did your breathing problems get better?
☐ Yes
☐ No

Use the lists below to identify the health care occupations you have had in the past 10 years.

Physician - Surgical

Physician - Anesthesiologist

Physician - Nonsurgical

Dentist

Podiatrist

Nurse - Anesthetist/Technician

Nurse - Operating Room

Nurse - Clinical (not Operating Room)

Nurse Practitioner or Physician Assistant

Nursing Assistant

Dental Assistant/Hygienist

Dental Lab Technician

Pharmacist/Clinical

Pharmacist/Lab Technician

Dialysis Technician

Medical Instruments Technician

Optometrist

Operating Room Technician (Endoscopy/bronchoscopy)

Clinical Laboratory Worker

(Medical Tech./Pathology/Histology/Cytology)

Phlebotomist

Research Laboratory Worker

Respiratory Therapist

Occupational Therapist

Physical Therapist

Speech Therapist/Audiology

Radiology Technician

Biomedical Engineer/Tech

Patient Care Asst. (transport, etc)

Social Worker/Psychologist

Dietician

Food Services Worker

Administrator

Environmental Safety

Office/Medical Records Worker

Ward Clerk

Radiology File Clerk

Facility/Maintenance Worker

Housekeeper (EMS)

Material Handler/Shipping

Medical Supply Tech. (SPD)

Ambulatory Surgical Care

Healthcare/Medical Tech.

Other - please specify

Occupation ______________________________ (you have had in the past 10 years)

What was your start date for this Occupation? ______________________________ (month-2 digits/year-4 digits (e.g. 06/2006))

What was your end date for this Occupation? ______________________________ (month-2 digits/year-4 digits (e.g. 06/2006))

Occupation ______________________________ (you have had in the past 10 years)

What was your start date for this Occupation? ______________________________ (month-2 digits/year-4 digits (e.g. 06/2006))

What was your end date for this Occupation? ______________________________ (month-2 digits/year-4 digits (e.g. 06/2006))

Occupation ______________________________ (you have had in the past 10 years)

What was your start date for this Occupation? ______________________________ (month-2 digits/year-4 digits (e.g. 06/2006))

What was your end date for this Occupation? ______________________________ (month-2 digits/year-4 digits (e.g. 06/2006))

Occupation ______________________________ (you have had in the past 10 years)

What was your start date for this Occupation? ______________________________ (month-2 digits/year-4 digits (e.g. 06/2006))

What was your end date for this Occupation?

____
(month-2 digits/year-4 digits (e.g. 06/2006))

Occupation

____
(you have had in the past 10 years)

What was your start date for this Occupation?

____
(month-2 digits/year-4 digits (e.g. 06/2006))

What was your end date for this Occupation?

____
(month-2 digits/year-4 digits (e.g. 06/2006))

Thank you for completing the questionnaire.
